# Colobine gut microbiome vulnerability to captivity: a meta-analysis of herbivorous primates

**DOI:** 10.1186/s42523-026-00600-6

**Published:** 2026-07-29

**Authors:** Marta Todó-Llorens, Nicola Rooney, Laura Peachey

**Affiliations:** University of Bristol, Bristol Veterinary School Langford House, Langford, North Somerset BS40 5DU UK

**Keywords:** Colobine primates, Gut microbiome, Captivity, Dysbiosis, Folivory, Foregut fermentation, Microbial diversity, Meta-analysis

## Abstract

**Background:**

Colobine monkey species have a multi-cavity forestomach adapted to a folivorous diet and foregut fermentation, making their gut microbiome essential for efficient digestion of plant fibre. In captivity, these species frequently experience gastrointestinal disorders associated with dietary changes and microbial dysbiosis. Understanding how their microbiome differs from that of other primates is therefore critical for improving health and husbandry. This study used a meta-analysis of global primate microbiome data to compare the faecal microbiome of colobines with those of other herbivorous primates, focusing on the effects of captivity.

**Results:**

Data from 16 studies involving 35 primate species and 7 primate families/subfamilies, comprising 1,690 faecal samples generated using 16 S rRNA amplicon sequencing, were re-analysed using standardised bioinformatic and compositional-statistical pipelines. Significant differences in microbial diversity and composition were observed among wild primates across families/subfamilies, diets, and fermentation strategies. Colobinae had a distinctive microbiome characterised by low Shannon diversity and high relative abundance of Firmicutes, particularly Ruminococcaceae and Lachnospiraceae, which were also prominent members of the wild colobinae core microbiome. Across all families/subfamilies and within colobinae, observed Shannon diversity was lower in captivity; however, multivariable mixed-effects models showed that alpha-diversity estimates were highly sensitive to study identity, species representation and methodological covariates. In contrast, beta-diversity and ALDEx2 analyses provided consistent evidence of captivity-associated compositional restructuring. Captive colobinae were enriched in Bacteroidetes and Spirochaetes, and families such as Prevotellaceae, Rikenellaceae, Methanobacteriaceae, Spirochaetaceae and Bacteroidaceae, whereas wild colobines were enriched in fibre-associated taxa, including Ruminococcaceae and Lachnospiraceae.

**Conclusions:**

Captivity alters the gut microbiota of colobine primates, affecting microbial diversity and core fibre-fermenting taxa important for gastrointestinal health. However, adjusted alpha-diversity models revealed substantial confounding by study, species, and methodological variables, indicating that diversity effects should be interpreted cautiously. Their specialised folivorous diet and foregut-fermenting physiology appear to produce a highly efficient yet fragile microbial ecosystem poorly adapted to ex-situ conditions. The strongest evidence for captivity-associated disruption was consistent alterations in faecal microbial composition, highlighting the need for diet formulations and microbiome-based health monitoring tailored to the unique digestive ecology of colobines to mitigate the high prevalence of gastrointestinal disorders in captivity.

**Supplementary Information:**

The online version contains supplementary material available at https://doi.org/10.1186/s42523-026-00600-6.

## Introduction

Specific gastrointestinal microbiomes may have facilitated the dietary adaptations in the different non-human primate species, especially in herbivores such as colobine primate species. Their specialised gut microbiome plays an essential role in plant digestion by effectively extracting energy from otherwise indigestible food sources [1, 2]. Herbivorous species follow two different fermentation strategies and can be divided into foregut and hindgut fermenters. Colobinae primates, commonly known as colobines, are a subfamily within the family Cercopithecidae, which includes two subfamilies, the cercopithecines and the colobines. Colobines are primarily arboreal and are distributed across both Africa and Asia [3]. They are exceptional among primates in possessing a digestive strategy analogous to that of ruminant mammals, relying on foregut fermentation within a multi-chambered stomach adapted to a folivorous diet [4]. The colobine forestomach is divided into the presaccus (absent in African colobines), the saccus gastricus, the tubus gastricus and the pars pylorica. In colobine monkeys, microbial fermentation occurs predominantly in the forestomach, with the saccus gastricus acting as the primary fermentation chamber and is complemented by secondary fermentation in the caecum and proximal colon. Captive colobines frequently experience gastrointestinal disorders, likely resulting from substantial differences in diet compared with wild populations, which can disrupt gut microbial homeostasis [1, 5–7]. A 19-year retrospective study of morbidity and mortality in colobines in UK zoos concluded that gastrointestinal disease and systemic infection were the principal causes of death [8]. Such disorders are thought to be closely linked to dietary imbalances and altered gut microbial composition.

The gut microbiome profoundly influences host energy metabolism, immune function, and intestinal homeostasis, and is particularly crucial in herbivorous mammals due to their dependence on microbial fermentation for energy acquisition from fibrous, plant-based diets [9–11]. Understanding the mechanisms underlying gut microbial homeostasis in colobines is therefore essential to improve captive management and prevent gastrointestinal disease in this vulnerable group.

The gut microbiome of colobine primates remains poorly characterised, and its differentiation from that of other herbivorous primates and response to captivity are not yet fully understood. No comprehensive studies have been conducted to systematically compare the gut microbiomes of colobine primates with those of a wide range of other herbivorous primates to investigate why colobines are especially vulnerable to gastrointestinal disease in captivity. Research on both African and Asian colobines has yielded valuable but sometimes inconsistent insights, likely due to variation in sampling, environmental context, and analytical methodologies [12–14]. In many wild colobine populations, the faecal microbiome is dominated by Firmicutes, particularly fibre-degrading families such as Ruminococcaceae and Lachnospiraceae, followed by variable proportions of Bacteroidetes and Verrucomicrobia [13, 15–18].

Captivity has been widely reported to alter the gut microbiome of non-human primates, often leading to decreased diversity, compositional shifts, and loss of functionally overlapping taxa [19–21]. In colobines, reported changes include enrichment of Bacteroidetes and human-associated genera such as *Prevotella* and *Bacteroides*, accompanied by a decline in fibre-degrading Firmicutes [7, 21–25]. Such patterns have been interpreted as indicators of “microbiome humanisation,” driven by differences in diet, microbial exposure, and increased hygiene between captive and wild environments [19]. Reduced alpha diversity in captive colobines has also been documented [7, 19, 21, 26], and is thought to reduce the ecological redundancy and stability of the gut ecosystem, lowering its capacity to buffer against perturbations or opportunistic pathogens [27, 28]. However, other studies have reported the opposite pattern, with captive individuals showing higher alpha diversity metrics than their wild conspecifics [22, 23, 29, 30]. Variation between study findings may be due to a range of technical and biological factors, and methodologies that account for this variation are vital for advancing understanding of this complex field.

To address current knowledge gaps, we conducted a meta-analysis comparing the faecal microbiome of colobine monkey species with those of other herbivorous primates, with a particular focus on the effects of captivity. By harmonising previously published datasets, we aimed to identify general patterns in microbiome composition and resilience among herbivorous primates and to test whether colobines are disproportionately affected by captivity. Meta-analyses are powerful tools to overcome the limitations and variability of individual studies. Combining data from previous studies effectively increases sample size and statistical power, providing more generalisable conclusions [31, 32]. However, the validity of such an approach depends on data quality and consistency. Accordingly, we re-analysed raw sequencing data from published studies using a unified bioinformatic and compositional-statistical pipeline to ensure methodological comparability [33]. Specifically, we compared: (1) wild individuals across all primate families and subfamilies included in this study; (2) wild foregut- *versus* hindgut-fermenting primates; (3) wild folivorous *versus* non-folivorous primates; (4) wild *versus* captive individuals across all families and subfamilies; and (5) wild *versus* captive colobines. We also characterised the core faecal microbiome of wild colobine populations. In addition, we examined key methodological variables (e.g., DNA extraction, sequencing platform, preservation method) to evaluate potential confounding effects on microbiome variation across studies.

## Methods

### Data collection

We searched studies between 2000 and 2020 using the databases CAB Abstracts, PubMed, Scopus, Web of Science-Core Collection, and Google Scholar. The search terms used were “*herbivorous primates microbiome*”, “*primate gut microbiome*”, “*primate foregut fermenter*”, and “*primate hindgut fermenter*”. Primate genus plus the term “*microbiome*”, and synonyms for each keyword were also used.

The following data was extracted from all articles matching the selection criteria: title, year published, common species name, scientific species name, species original habitat (country), site (environmental condition: captive or wild), species fermentation type (foregut or hindgut), diet (folivore or non-folivore), gastrointestinal sample location, gastrointestinal health status and general health status, age and sex of study individuals, number of samples, sequencing analysis, instrument and primers used, stabilisation method, storage and DNA extraction method [see Additional file 1]. The primate families-subfamilies included in this study were Alouatinnae, Cercopithecinae, Colobinae, Homininae, Indriidae, Lemuridae and Lepilemuriadae. Folivore family-subfamily groups included in this study were Colobinae, Alouattinae, Indriidae and Lepilemuridae, and non-exclusive folivores or non-folivores were Cercopithecinae, Homininae and Lemuridae.

### Bioinformatics

Raw 16 S rRNA amplicon sequencing data and associated metadata were downloaded from original database sources, such as the NCBI GenBank database, the Sequence Read Archive (SRA), the European Nucleotide Archive (ENA), and the European Bioinformatics Institute (EBI), or were directly provided by the corresponding authors when the sequencing data were not publicly available. Most studies targeted the V4 region of the 16 S rRNA gene, although some datasets used V1–V2, V1–V3, or V3–V4 amplicons. The QIIME2 software (2020.8) was used for quality control, feature table construction, and classification [34]. Studies were classified and grouped by sequencing platform and read type (single-end or paired-end). Sequencing data in the CASAVA 1.8 demultiplexed format (single-end or paired-end) were imported into QIIME2 artifacts. Quality filtering and feature table construction were carried out using the DADA2 pipeline implemented in the q2-dada2 plugin [35]. This step generates QIIME2 artifacts containing a feature table with the number of sequences (reads) per sample and the corresponding unique sequence identifiers (amplicon sequence variants, ASVs). The DADA2 denoising process requires specification of two parameters: --p-trim-left (the number of bases trimmed from the 5′ end) and --p-trunc-len (the position at which sequences are truncated based on quality). Truncation and trimming parameters were optimised separately for each analysis group based on quality score plots visualised from the demultiplexed data. This ensured that low-quality bases were removed while maximising retention of high-quality reads across datasets that varied in sequencing technology and read length. The DADA2 pipeline inferred amplicon sequence variants (ASVs), which were retained as the primary sequence-resolution units throughout QIIME2 processing. The resulting feature table and representative sequences provided sequence counts per sample and feature-to-sequence mappings. A phylogenetic tree was constructed using the align-to-tree-mafft-fasttree pipeline, which first aligned sequences with MAFFT and then inferred a phylogeny using FastTree through the q2-phylogeny plugin [36, 37]. Initial exploratory alpha- and beta-diversity outputs were generated in QIIME2 using the q2-diversity plugin after rarefaction within technically comparable sequencing groups. Rarefaction depths were selected following inspection of the feature table artifacts (table.qzv) to maximise retention of samples while excluding low-depth libraries within each dataset. However, the statistical analyses reported in this study were conducted in R after importing the filtered ASV feature tables, taxonomy tables, and metadata into phyloseq. The q2-feature-classifier Naive Bayes classifier was trained for each analysis group against the Silva 132 database to classify the representative sequences’ taxonomies, yielding the taxonomy Tables [38, 39]. The resulting feature tables (table.qza), taxonomy tables, and the metadata were merged using the q2-feature-table plugin. Finally, merged artifacts (feature table and taxonomy table) were exported for downstream analysis in R using the QIIME tools export command. The feature table (BIOM format) was converted to a tab-delimited text file (.txt), and the taxonomy table to a comma-separated file (.csv), to facilitate compatibility with R-based analytical pipelines.

### Statistical analysis

The ASV feature table and the taxonomy table were imported into R (version 4.1.1), along with the metadata. The R-packages used for the statistical analysis were “phyloseq”, “vegan”, “microbiome” and “ALDEx2”. For the analysis, only faecal samples from healthy individuals were selected (*N* = 1690) and the samples, and metadata were organised into four analytical groups (using phyloseq::subset_samples) in R and analysed accordingly: (1) Samples from wild individuals of all primate families; (2) Samples from wild and captive individuals from all primate families for comparison of wild *versus* captive across all families; (3) Wild and captive samples from the subfamily colobinae for comparison of wild *versus* captive colobines; and (4) Samples from wild colobines only for analysis of the core colobine microbiome. Following import and metadata subsetting, ASVs/features with zero counts were removed, and the phyloseq object was created [40]. Singletons and doubletons (features with one or two reads mapped in one or more samples), ambiguous taxa, or those not classified at the Phylum level were removed. Because datasets differed in sequencing region, platform, and taxonomic resolution, ASV-level identifiers were not directly comparable across studies. Therefore, feature counts were aggregated to a higher taxonomic rank, at family level, to enable standardised cross-study comparative analyses. Taxa assigned to chloroplast, mitochondria, or other non-bacterial origins (e.g., plant, fungal, or protozoan sequences) were removed following taxonomic inspection of the aggregated dataset, as they represent common environmental contaminants or host-derived sequences in faecal 16 S rRNA gene datasets. Non-bacterial taxa identified and excluded at the family level were: *Ammopiptanthus mongolicus*, Ambiguous taxa, *Anemone flaccida*, *Eimeria praecox*, *Euglena deses*, Mitochondria, *Millettia pinnata*, *Pardosa pseudoannulata*, *Quercus robur*,* Solanum pennellii*, *Tanaecium tetragonolobum*, *Phytophthora lateralis* MPF4, *Virgulinella fragilis*, *Vismia ferruginea*, *Ziziphus jujuba*, and *Bryum argenteum* var. *argenteum*.

The phyloseq object was transformed to relative abundance, and the relative abundance at the phylum level was summarised for wild primate samples by family/subfamily.

Alpha diversity was calculated using the Shannon diversity index on relative abundance-transformed data aggregated at the family level. Initial descriptive comparisons among wild primate families/subfamilies were retained to characterise broad family-level diversity patterns. Because these exploratory comparisons did not meet assumptions of normality and homogeneity of variance, Kruskal-Wallis tests were used, followed by pairwise Wilcoxon rank-sum tests with Benjamini-Hochberg correction where appropriate.

To address potential confounding among biological, technical, and study-level variables, subsequent alpha-diversity analyses were conducted using multivariable linear mixed-effects models implemented with the lme4 and lmerTest packages [41]. Per-sample read depth was extracted from the filtered count table, log10-transformed, and included as a fixed effect because Shannon diversity can remain sensitive to sequencing depth through differences in taxon detection. Study identity was included as a random intercept to account for non-independence among samples from the same study.

For wild samples across all primate families/subfamilies, models assessed the effects of fermentation strategy and diet while adjusting for sequencing instrument, sample stabilisation method, storage method, DNA extraction protocol, log-transformed read depth, and study identity. Because fermentation strategy and diet partially overlapped biologically, an initial model including both predictors was evaluated, followed by separate adjusted models for fermentation strategy and diet to facilitate interpretation. For site (wild *versus* captive) comparisons across all families/subfamilies, alpha diversity was analysed using mixed-effects models that included site, log-transformed read depth, and study identity, with additional models incorporating family/subfamily and site × family/subfamily interactions to assess whether captivity-associated patterns differed among taxonomic groups. Additional diagnostic models were fitted sequentially to evaluate whether the estimated effect of captivity changed after accounting for family/subfamily composition, study identity, and technical covariates.

For the colobine-only analysis, alpha diversity was compared between wild and captive samples using mixed-effects models including site, log-transformed read depth, available technical covariates, and study identity as a random intercept. Additional diagnostic models were used to assess whether the estimated wild–captive difference was sensitive to primate species, study identity, and technical covariates. Model significance was assessed using Type III ANOVA with Satterthwaite approximation for degrees of freedom, and estimated marginal means and contrasts were calculated using emmeans where appropriate.

For analysis of beta diversity, which quantifies the dissimilarity of the taxonomic composition between two samples, a compositional approach was used [42], and every data subset was transformed using the centered log-ratio (CLR). Beta diversity was assessed using Aitchison distance (Euclidean distance on CLR-transformed data). Principal Component Analysis (PCA) was then applied to the Aitchison distance matrix to visualise clustering and differences among samples. For ordination figures, both PC1-PC2 and PC2-PC3 axes were examined, and axis orientation was kept consistent across comparable plots to facilitate interpretation.

Differences in community composition were tested using Permutational Multivariate Analysis of Variance Using Distance Matrices (PERMANOVA) [43] with the adonis2 function in the vegan package [44]. Multivariable PERMANOVA models were fitted using by = “margin”, allowing each fixed-effect predictor to be tested while controlling for the other fixed effects in the model. PERMANOVA does not directly include random intercepts; therefore, study identity was not modelled as a random effect in these analyses. Where possible, sensitivity analyses were performed using study identity as permutation strata to account partially for study-level structure.

For wild samples across all families/subfamilies, separate adjusted PERMANOVA models were fitted for diet and fermentation strategy because these variables partially overlapped biologically. These models included diet or fermentation strategy as the main fixed effect and sequencing instrument, stabilisation method, storage method, and DNA extraction protocol as additional fixed-effect covariates. For wild-*versus*-captive (site) comparisons across all families/subfamilies, site was included as the main fixed effect, with technical covariates as additional fixed effects; an additional model also included diet and fermentation strategy as fixed effects. For the colobine-only wild *versus* captive comparison, site was included as the main fixed effect, with available technical covariates included as fixed-effect covariates, and study identity was evaluated as permutation strata where possible.

Homogeneity of multivariate dispersion was assessed using betadisper and Permutational Analysis of Multivariate Dispersion (PERMDISP) [44, 45]. Because significant PERMDISP results indicate that PERMANOVA effects may reflect differences in dispersion as well as centroid location, PERMANOVA and PERMDISP outputs were interpreted together for each subset. Full PERMANOVA and PERMDISP outputs are provided in the additional files.

Differential-abundance analyses were conducted at both phylum and family levels using the ALDEx2 package [46], which accounts for compositionality and sampling variation using a Dirichlet-multinomial Monte Carlo approach [47]. Analyses were performed on count data aggregated to the relevant taxonomic rank, with 295 Monte Carlo instances and denom = “all”.

For wild-only analyses across all primate families/subfamilies, ALDEx2 generalised linear models were used to test the effects of diet and fermentation strategy separately, because these variables partially overlapped biologically. Diet models included diet as the main predictor and sequencing instrument, stabilisation method, storage method, and DNA extraction protocol as fixed-effect covariates. Fermentation models included fermentation strategy as the main predictor with the same technical covariates.

For wild *versus* captive comparisons across all families/subfamilies, ALDEx2 fixed-effect GLMs were used to test the effect of site while accounting for available biological and technical covariates. For the colobine-only wild *versus* captive analysis, site was tested while adjusting for available technical covariates, including sequencing instrument, stabilisation method, storage method, and DNA extraction protocol where estimable. Model matrices were inspected for rank deficiency, and non-estimable covariate columns were dropped where necessary.

Because ALDEx2 does not directly support random effects, additional sensitivity analyses were performed by extracting expected CLR values from ALDEx2 and fitting taxon-wise linear mixed-effects models with study identity as a random intercept. These mixed models were used only as sensitivity analyses, while the ALDEx2 GLM outputs were retained as the primary differential-abundance results.

Significant taxa were identified using Benjamini-Hochberg adjusted p-values from the ALDEx2 GLM outputs, with adjusted *p* < 0.05 considered significant. Direction of enrichment was determined from the GLM coefficient estimates, with positive estimates indicating enrichment in the non-reference group. Significant results were visualised using bar plots generated with ggplot2 [48].

The core microbiome was analysed in wild samples of colobine monkeys, with a minimum sampling prevalence threshold of 90% and a minimum abundance threshold of 1%, using the phyloseq package. To visualise it, a heatmap plot representing the core with absolute counts was created. Lastly, the packages eulerr [49] and microbiome [50] were applied to create a Venn diagram following core microbiome results.

## Results

The 1690 included samples represented 7 primate families-subfamilies, and 35 primate species, including 12 colobines species [see Additional file 1]. The total number of raw reads before data processing for each analysis subset was: (a) 53,022,076 for wild samples across all primate families and subfamilies, (b) 60,121,507 for wild *versus* captive samples across all primate families and subfamilies, (c) 10,175,715 for wild *versus* captive Colobinae samples, and (d) 8,284,365 for wild Colobinae samples. After quality filtering, the corresponding reads were 52,217,593, 58,942,959, 9,780,487 and 8,255,948, respectively. Following taxonomic aggregation at the family level, reads assigned to non-bacterial taxa were removed, and the final read counts were 51,945,552, 58,653,884, 9,764,125, and 8,239,876, respectively.

### Faecal microbiome composition and diversity differ among wild primate families and subfamilies using a meta-analysis approach

**Fig. 1:**
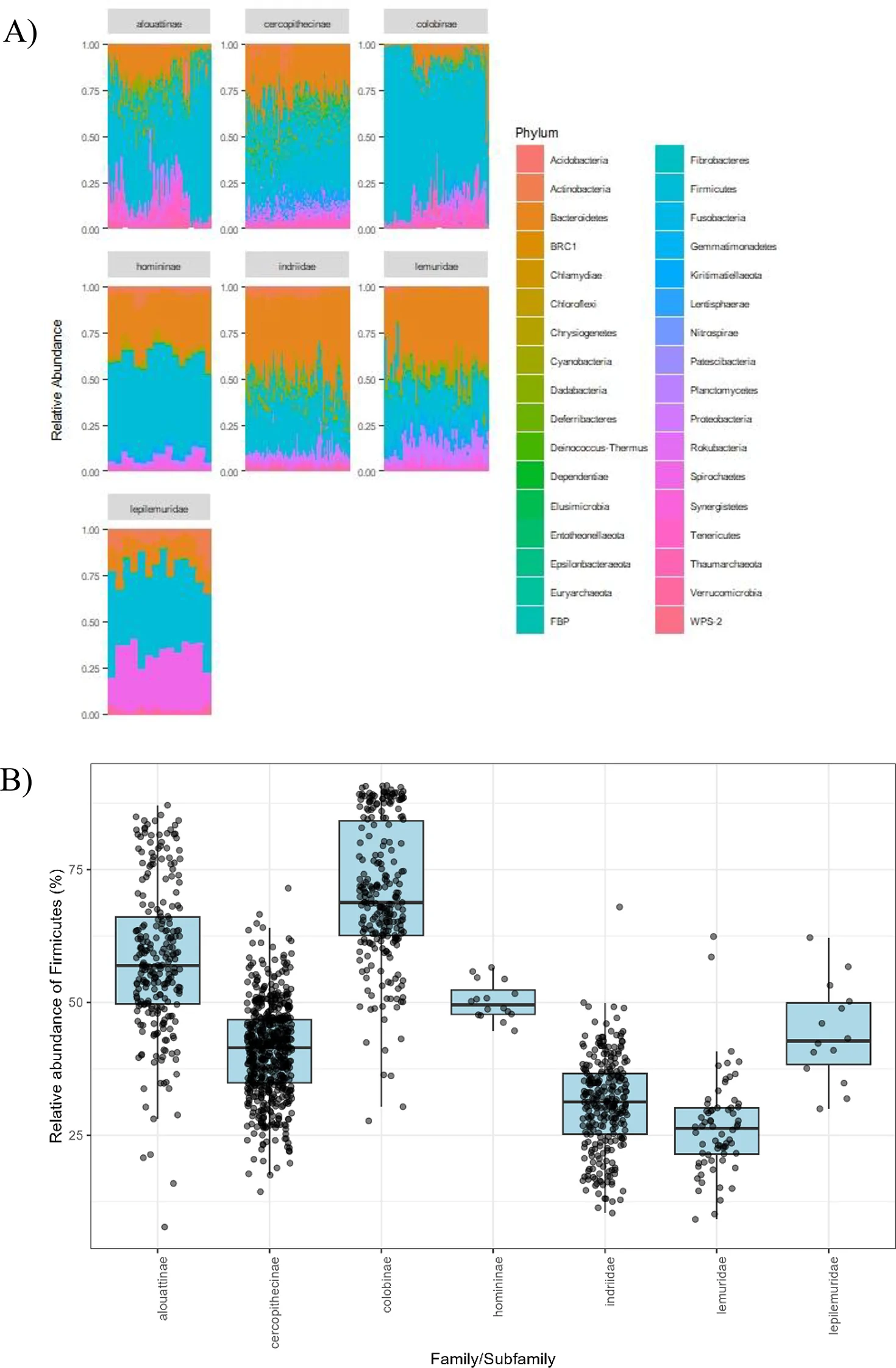

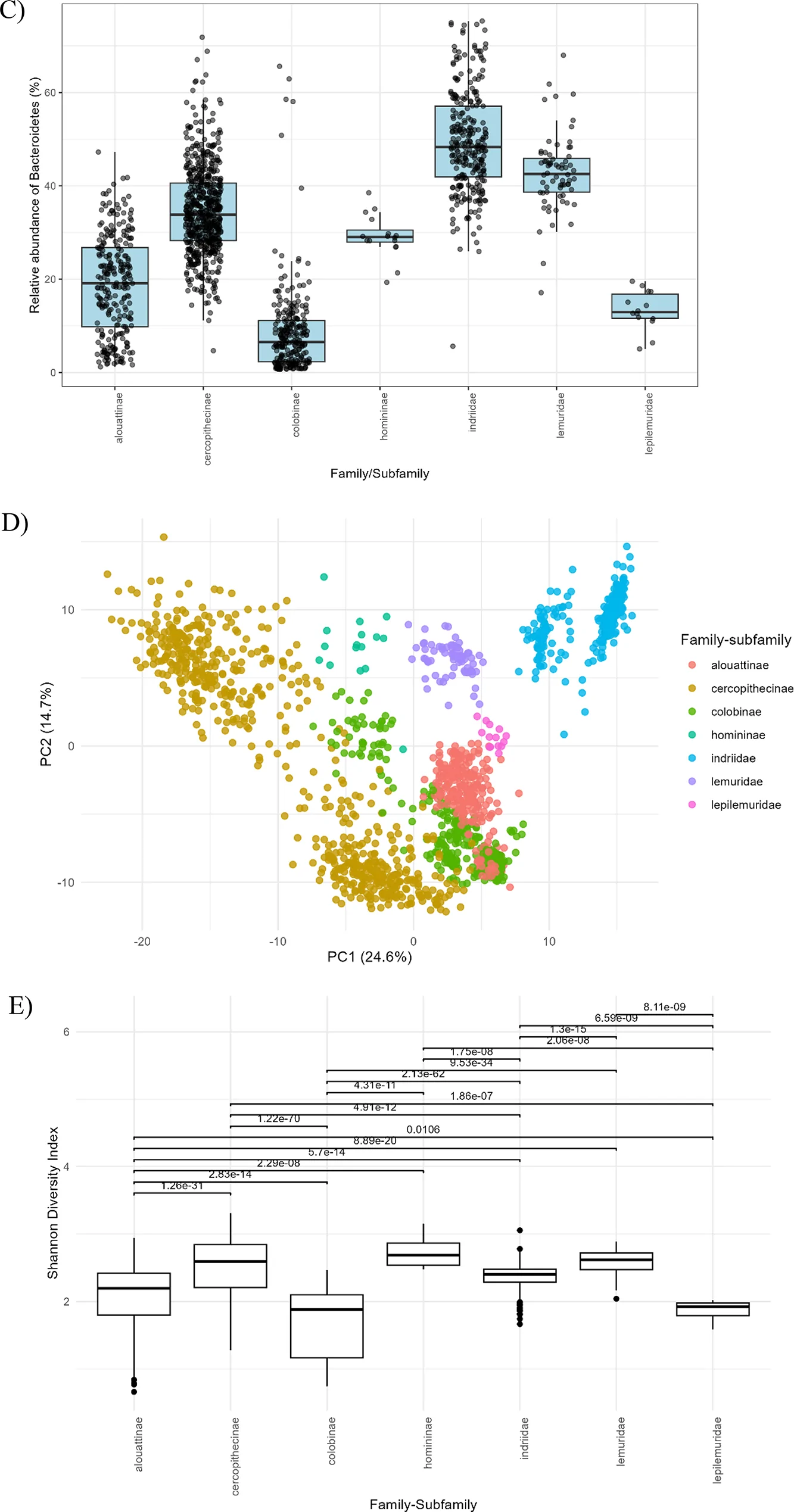
Gut microbiome composition and diversity across wild primate families and subfamilies. (**A**) Relative abundance of bacterial phyla in wild individuals, summarised by primate family–subfamily. Each bar represents a single sample, coloured by phylum. (**B**–**C**) Relative abundance of Firmicutes (**B**) and Bacteroidetes (**C**) across wild primate families–subfamilies. Boxplots show median, interquartile range, and range; points represent individual samples. (**D**) Principal component analysis (PCA) based on Aitchison distances of CLR-transformed phylum-level data, illustrating differences in gut microbial community composition among families–subfamilies. (**E**) Shannon diversity index across wild primate families–subfamilies. Boxplots show median and interquartile range. Horizontal bars indicate significant pairwise comparisons based on Wilcoxon rank-sum tests with Benjamini–Hochberg correction

In wild individuals, we detected thirty-four main phyla. An additional file shows the mean relative abundance (%) of these phyla [see Additional file 2]. Firmicutes was the most abundant phylum in all wild families-subfamilies, except in the Indriidae and Lemuridae families, and its relative abundance varied among groups. Bacteroidetes was the next most abundant phyla followed by Proteobacteria and Actinobacteria. The relative abundance of Firmicutes was highest in the Colobinae subfamily (77.7%), followed by the Alouattinae subfamily (61.97%), and the Homininae family (50.73%; Fig. 1A-B). Whereas Bacteroidetes was the most abundant phylum in the Indriidae and Lemuridae families, at 50.66% and 43.61%, respectively (Fig. 1A and C). Kruskal-Wallis tests showed that the abundance of the top phyla (Firmicutes, Bacteroidetes, Proteobacteria and Actinobacteria) varied significantly between all the primate families-subfamilies (χ^2^ > 373, *p* < 2.2e-16).

The PERMANOVA test on the Aitchison dissimilarity matrix showed significant differences in the beta diversity of bacterial communities between families-subfamilies in the wild samples (pseudo-F = 148.97, R² = 0.381, *p* = 0.001) (Fig. 1D). There was a significant difference in the dispersion of the wild family-subfamily groups (*p* < 2.2e-16), so the Permutational Analysis of Multivariate Dispersion (PERMDISP) test was performed, which confirmed the significant differences in community composition between families-subfamilies (F = 171.62, *p* = 0.001).

When the Shannon index was compared between wild different sample subsets, using the Kruskal-Wallis test, significant differences were seen among family-subfamily groups (χ^2^ = 531.34, *p* < 2.2e-16; Fig. 1E). Pairwise Wilcoxon tests showed that Colobines had a significantly lower Shannon index compared to the other families-subfamilies, except for the Lepilemuridae family (*p* < 0.01, Fig. 1E).

### Fermentation strategy and diet are associated with microbiome variation in wild primates

**Fig. 2:**
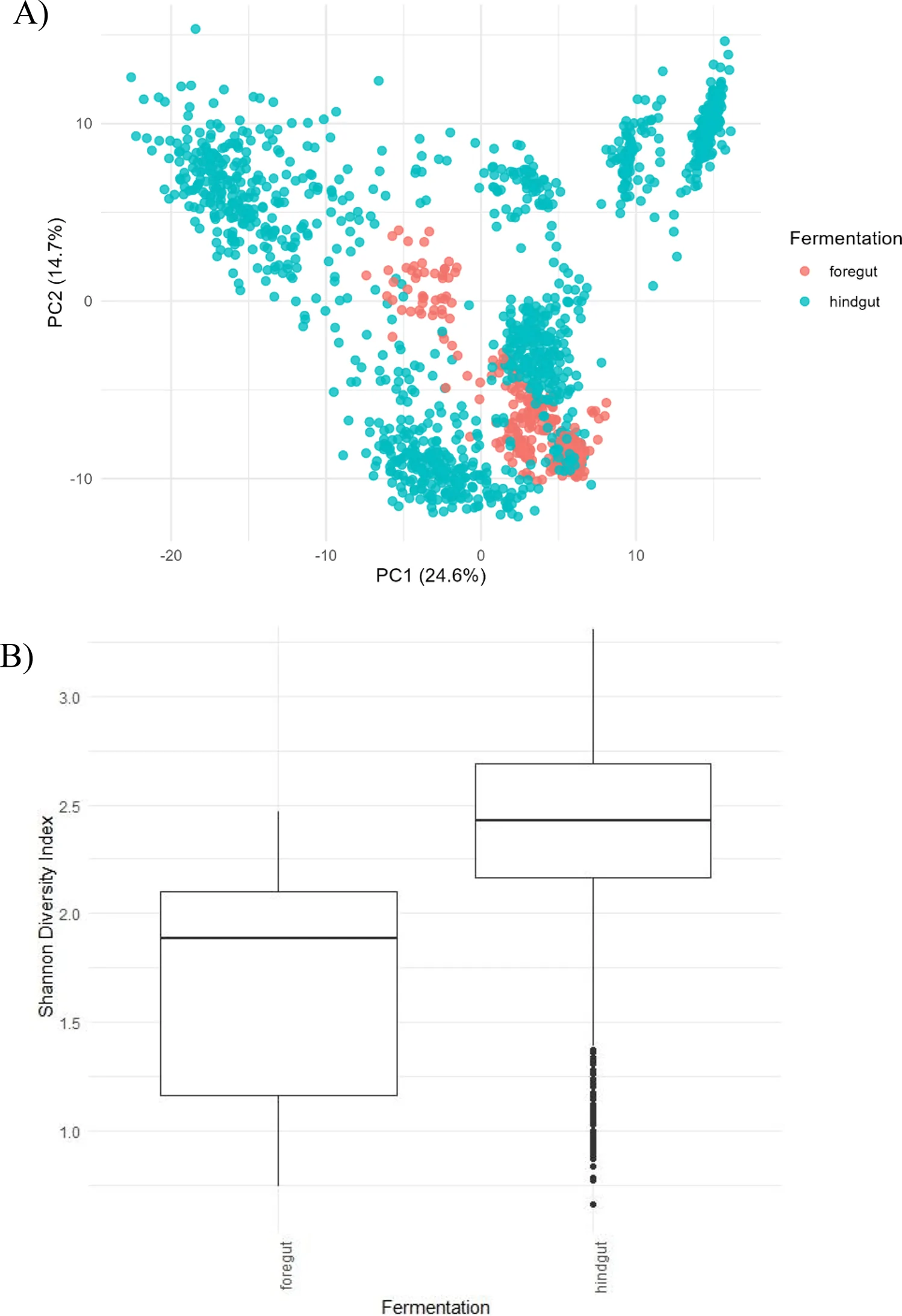

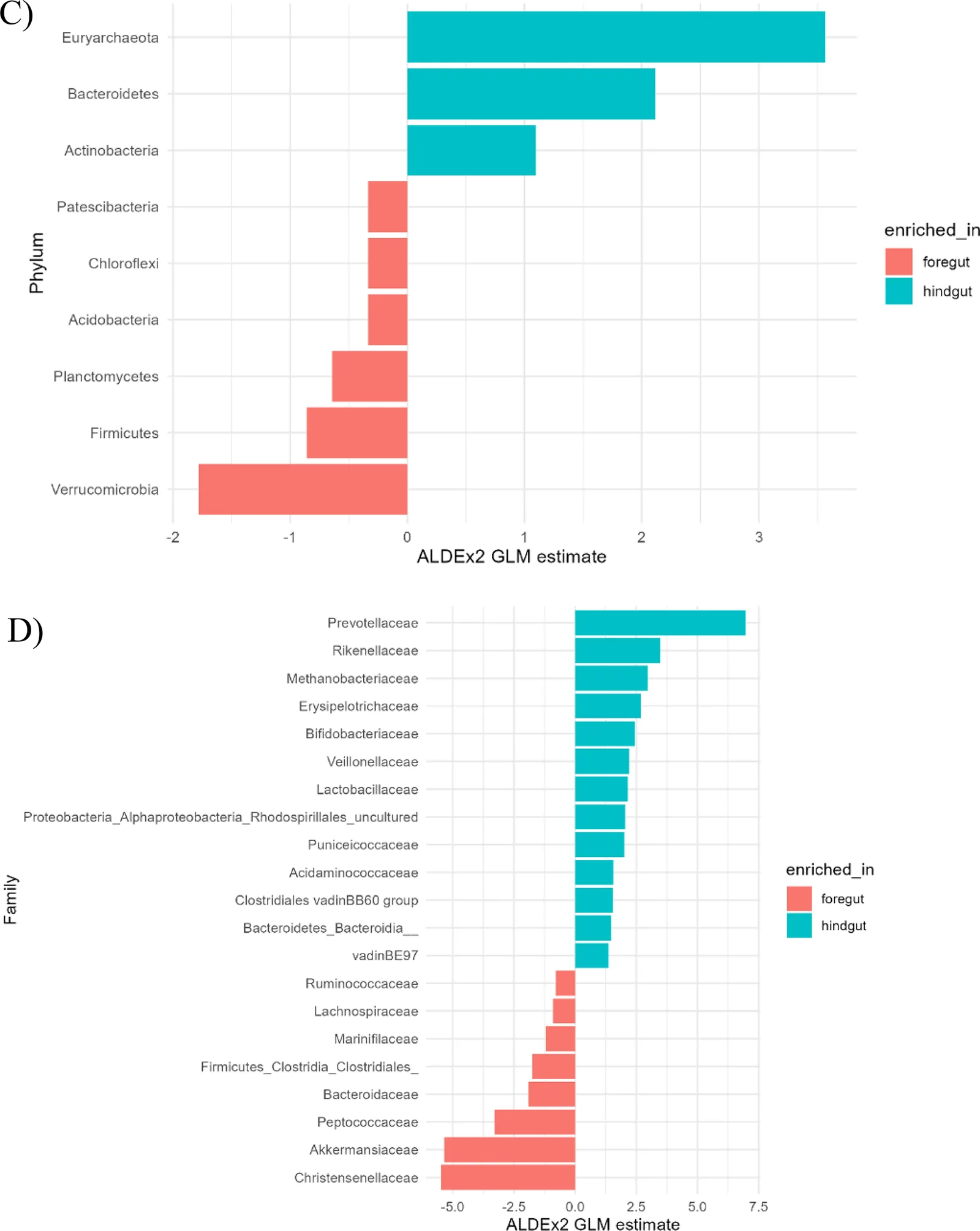
Gut microbiome differences between foregut- and hindgut-fermenting primates. (**A**) PCA of Aitchison distances calculated from centred log-ratio (CLR)-transformed family-level data for wild primate samples, coloured by fermentation strategy (foregut vs. hindgut). (**B**) Comparison of Shannon diversity indices between foregut- and hindgut-fermenting primates. Boxes represent interquartile ranges, centre lines indicate medians, and points represent individual samples. (**C**) ALDEx2 differential-abundance analysis at the phylum level, showing taxa with the largest effect sizes differentiating foregut- and hindgut-fermenting primates. Bars represent effect sizes, and only taxa with significant differences after Benjamini–Hochberg correction (BH-adjusted *p* < 0.05) are shown. (**D**) ALDEx2 differential-abundance analysis at the family level

**Fig. 3:**
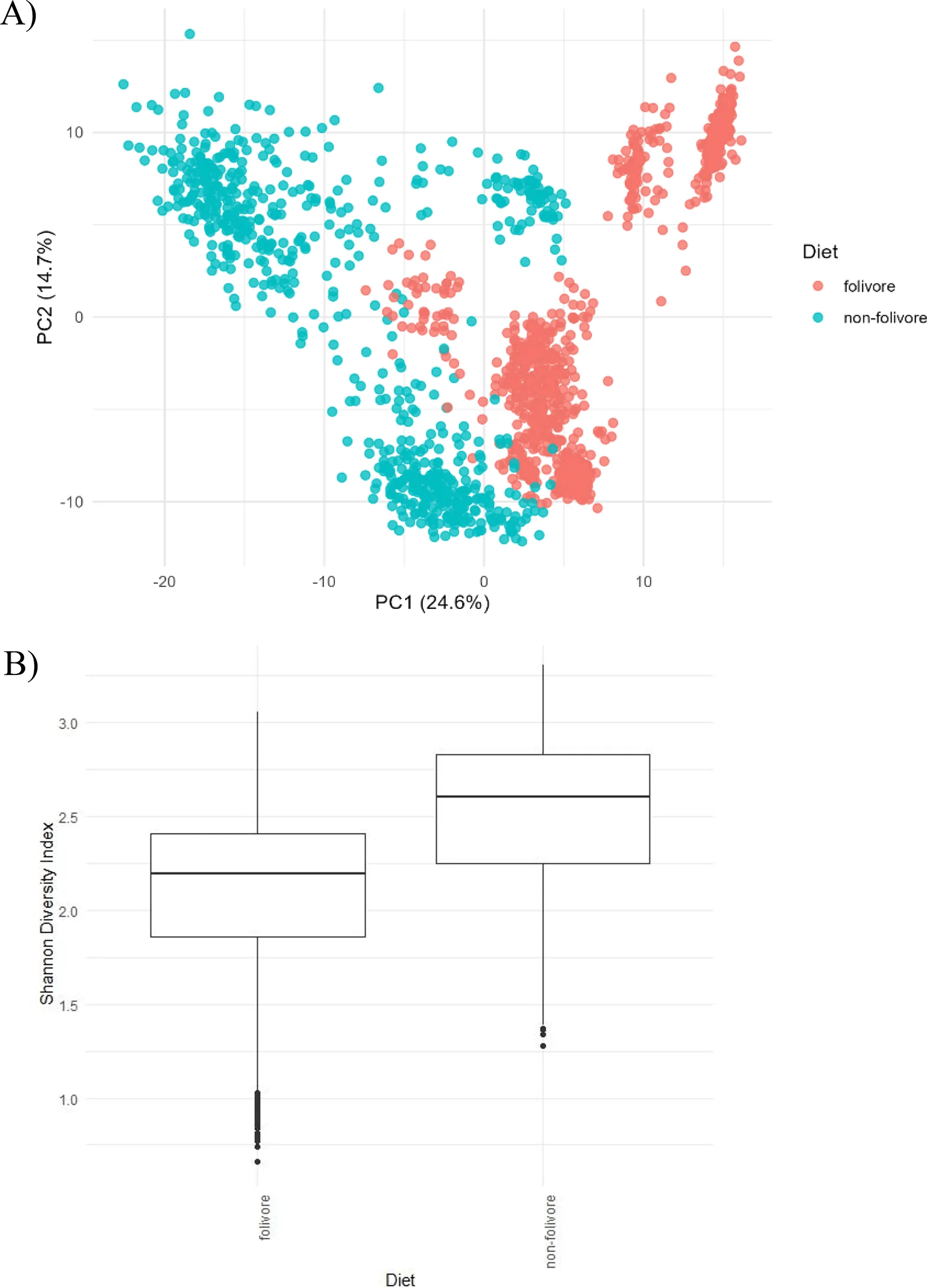

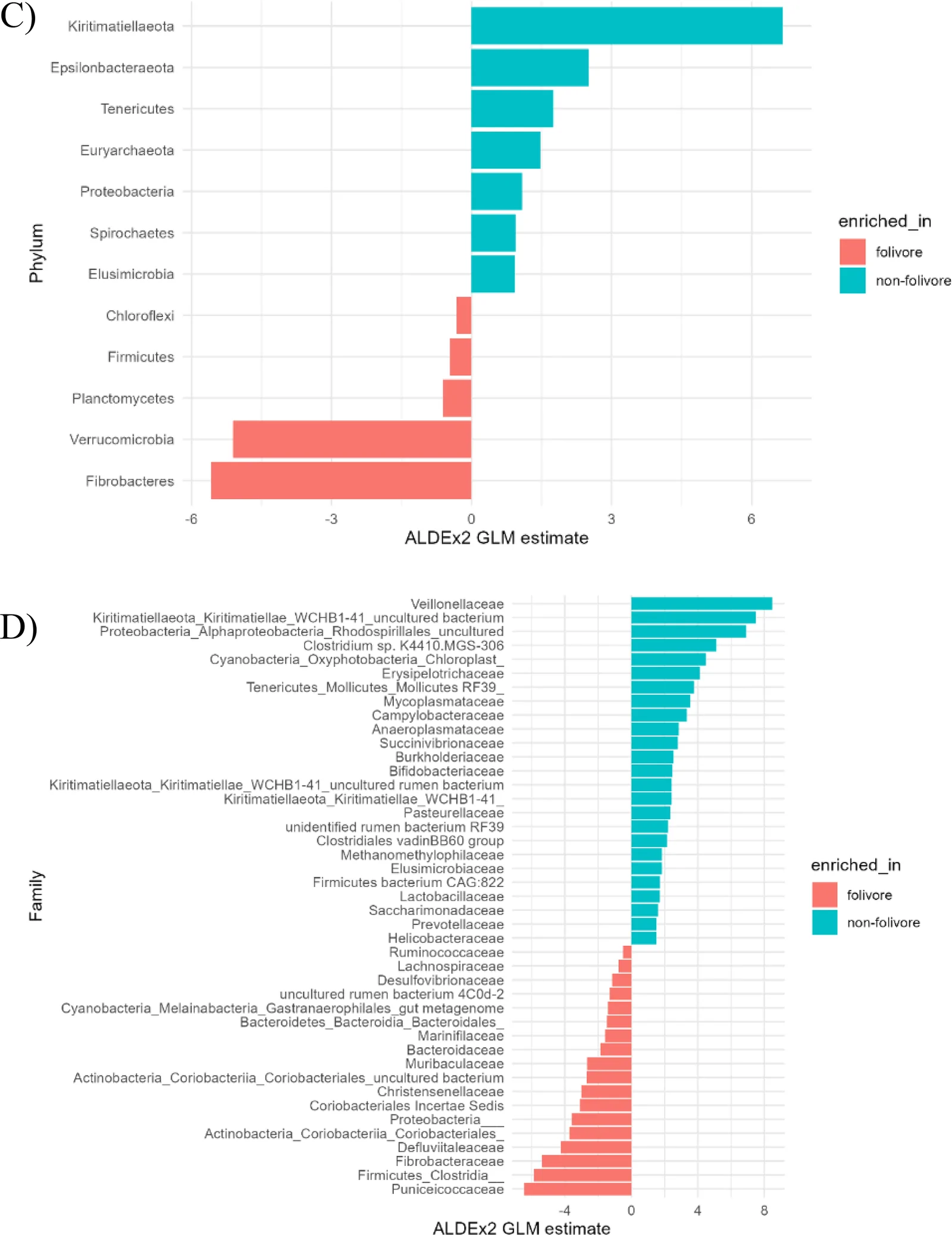
Gut microbiome differences between folivorous and non-folivorous primates. (**A**) PCA of Aitchison distances calculated from centred log-ratio (CLR) transformed family-level data for wild primate samples, coloured by dietary category (folivore vs. non-folivore). Ordination illustrates clear compositional separation between dietary groups. (**B**) Comparison of Shannon diversity indices between folivorous and non-folivorous primates, showing significantly lower alpha diversity in folivores. Boxes represent interquartile ranges, centre lines indicate medians, and points represent individual samples. (**C**) ALDEx2 differential-abundance analysis at the phylum level, showing taxa with the largest effect sizes differentiating folivorous and non-folivorous primates. Bars represent effect sizes, and only taxa with significant differences after Benjamini–Hochberg correction (BH-adjusted *p* < 0.05) are shown. (**D**) ALDEx2 differential-abundance analysis at the family level

Exploratory comparisons using the Shannon diversity index showed lower alpha diversity in foregut fermenters relative to hindgut fermenters (Kruskal-Wallis χ² = 325.28, *p* < 2.2e-16; Fig. 2B), and lower diversity in folivores compared to non-folivores (χ² = 346.12, *p* < 2.2e-16; Fig. 3B).

To account for potential methodological and study-level confounding, alpha diversity was subsequently analysed using multivariable linear mixed-effects models including fermentation strategy, diet, sequencing instrument, stabilisation method, storage method, DNA extraction protocol, sequencing depth, and study identity as a random effect. In the combined model, diet remained a significant predictor of Shannon diversity after adjustment for technical covariates and study identity (F = 88.38, *p* < 2e-16), whereas fermentation strategy was no longer significant (F = 0.103, *p* = 0.748). Log-transformed sequencing depth also remained a significant predictor of Shannon diversity (F = 69.80, *p* < 2e-16). Because fermentation strategy and diet partially overlapped biologically, additional adjusted models were run separately for each variable to facilitate the interpretation of their independent associations with alpha diversity. In the adjusted fermentation-only model, hindgut fermenters showed significantly higher Shannon diversity than foregut fermenters after controlling for technical covariates, sequencing depth, and study identity (estimate = + 0.099, F = 7.69, *p* = 0.0056). Similarly, the adjusted diet-only model showed significantly higher Shannon diversity in non-folivores relative to folivores (estimate = + 0.306, F = 96.49, *p* < 2e-16).

Beta diversity analyses based on Aitchison distance of CLR-transformed family-level data differed significantly between foregut and hindgut fermenters in wild populations (Fig. 2A; see Additional file 5 Fig. S1). In the adjusted PERMANOVA model including fermentation strategy and technical covariates as fixed effects, fermentation strategy remained a significant predictor of microbiome composition (pseudo-F = 24.96, R² = 0.0069, *p* = 0.001). Storage method also explained a substantial proportion of compositional variation (pseudo-F = 177.55, R² = 0.0488, *p* = 0.001), whereas stabilisation method had a smaller but significant effect (pseudo-F = 6.39, R² = 0.0018, *p* = 0.001). Multivariate dispersion also differed significantly between foregut and hindgut fermenters (PERMDISP: F = 463.36, *p* = 0.001), indicating unequal within-group variability. Consequently, the observed PERMANOVA differences likely reflect both differences in centroid location and differences in within-group dispersion.

Between folivores and non-folivores primates, beta diversity analyses showed clear compositional separation in wild populations (Fig. 3A; see Additional file 5 Fig. S2). In the adjusted PERMANOVA model including diet and technical covariates as fixed effects, diet explained a significant proportion of microbiome compositional variation (pseudo-F = 74.89, R² = 0.0199, *p* = 0.001). Storage method also explained a significant proportion of variation (pseudo-F = 183.60, R² = 0.0488, *p* = 0.001), whereas stabilisation method showed a smaller but significant effect (pseudo-F = 6.60, R² = 0.0018, *p* = 0.001). Homogeneity of dispersion differed significantly between dietary groups (PERMDISP: F = 163.0, *p* = 0.001), indicating greater within-group heterogeneity in one or both diet categories. Therefore, the observed PERMANOVA differences likely reflect both differences in community composition and differences in multivariate dispersion.

ALDEx2 differential abundance analysis identified clear compositional differences between foregut- and hindgut-fermenting primates at both phylum and family levels in wild populations only. Results are summarised here for taxa showing the largest effect sizes, while the full results are provided in the additional files [see Additional files 3 and 4]. At the phylum level, foregut fermenters showed higher relative abundance of Verrucomicrobia, Firmicutes, Planctomycetes, Acidobacteria, Chloroflexi, and Patescibacteria, whereas hindgut fermenters were enriched in Euryarchaeota, Bacteroidetes and Actinobacteria (BH-adjusted *p* < 0.05, where BH represents the Benhamin-Hochberg adjusted within-condition p-value generated by ALDEx2; Fig. 2C). At the family level, several taxa were significantly enriched in foregut fermenters, including Christensenellaceae, Akkermansiaceae, Peptococcaceae, Bacteroidaceae, Lachnospiraceae, and Ruminococcaceae (BH-adjusted *p* < 0.05; Fig. 2D). In contrast, hindgut fermenters showed higher relative abundance of Prevotellaceae, Rikenellaceae, Methanobacteriaceae, Erysipelotrichaceae, Bifidobacteriaceae, Veillonellaceae and Lactobacillaceae.

ALDEx2 differential abundance analysis also identified marked compositional differences between folivorous and non-folivorous primates at both phylum and family levels in wild populations only. Results are summarised here for taxa showing the largest effect sizes, with the results available in the additional files [see Additional files 6 and 7]. At the phylum level, folivorous primates showed higher relative abundance of Fibrobacteres, Verrucomicrobia, Planctomycetes, Firmicutes, and Chloroflexi, whereas non-folivorous primates were enriched in Kiritimatiellaeota, Epsilonbacteraeota, Tenericutes, Proteobacteria, and Spirochaetes (BH-adjusted *p* < 0.05; Fig. 3C). At the family level, several taxa were significantly enriched in folivores, including Puniceicoccaceae, Fibrobacteraceae, Christensenellaceae, Muribaculaceae, Bacteroidaceae, Desulfovibrionaceae, Lachnospiraceae, and Ruminococcaceae (BH-adjusted *p* < 0.05; Fig. 3D). In contrast, non-folivorous primates showed higher relative abundance of Veillonellaceae, Erysipelotrichaceae, Mycoplasmataceae, Campylobacteraceae, Succinivibrionaceae, Bifidobacteriaceae, Pasteurellaceae and Prevotellaceae.

### Captivity is associated with microbiome restructuring across primate families and subfamilies

**Fig. 4:**
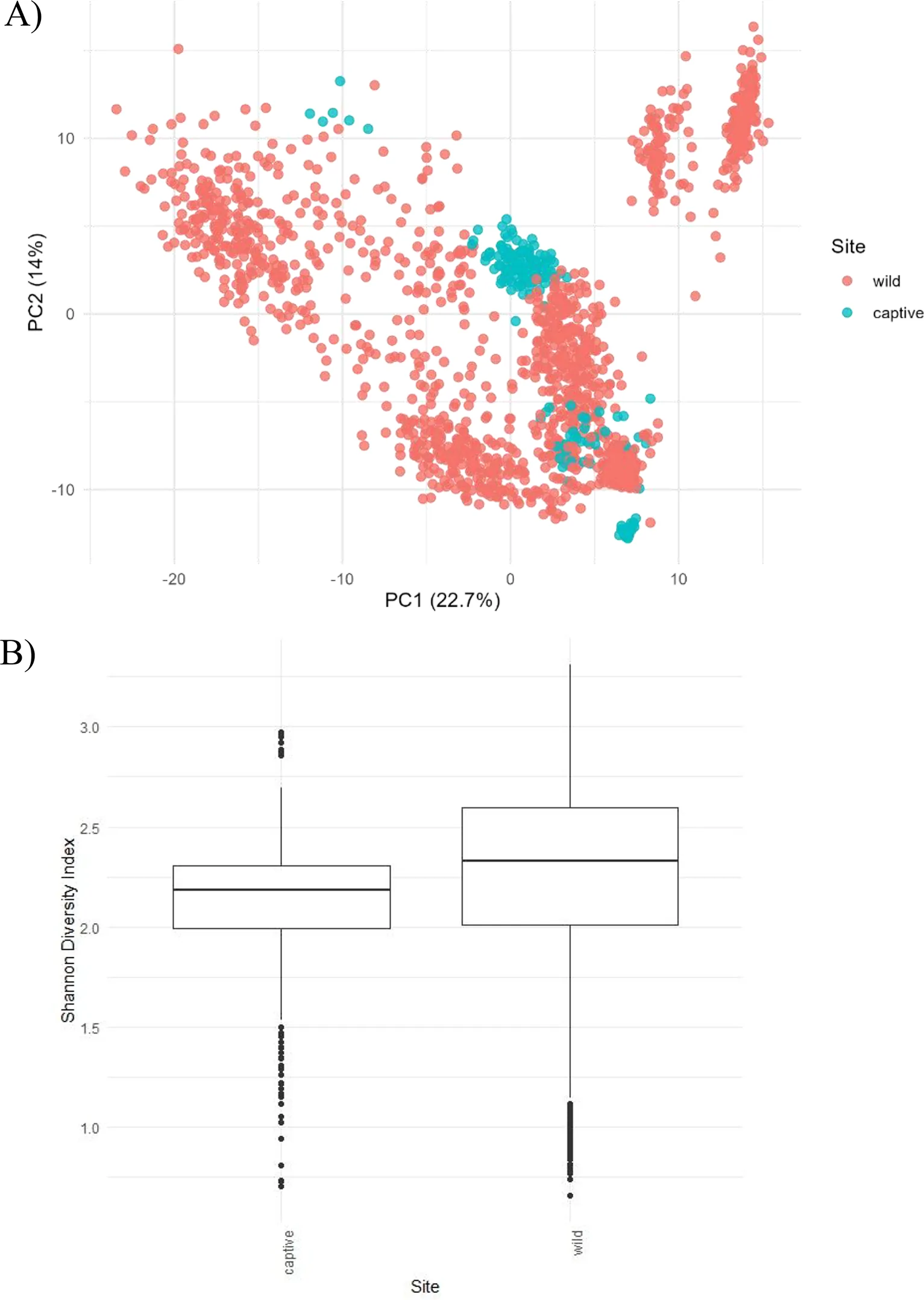

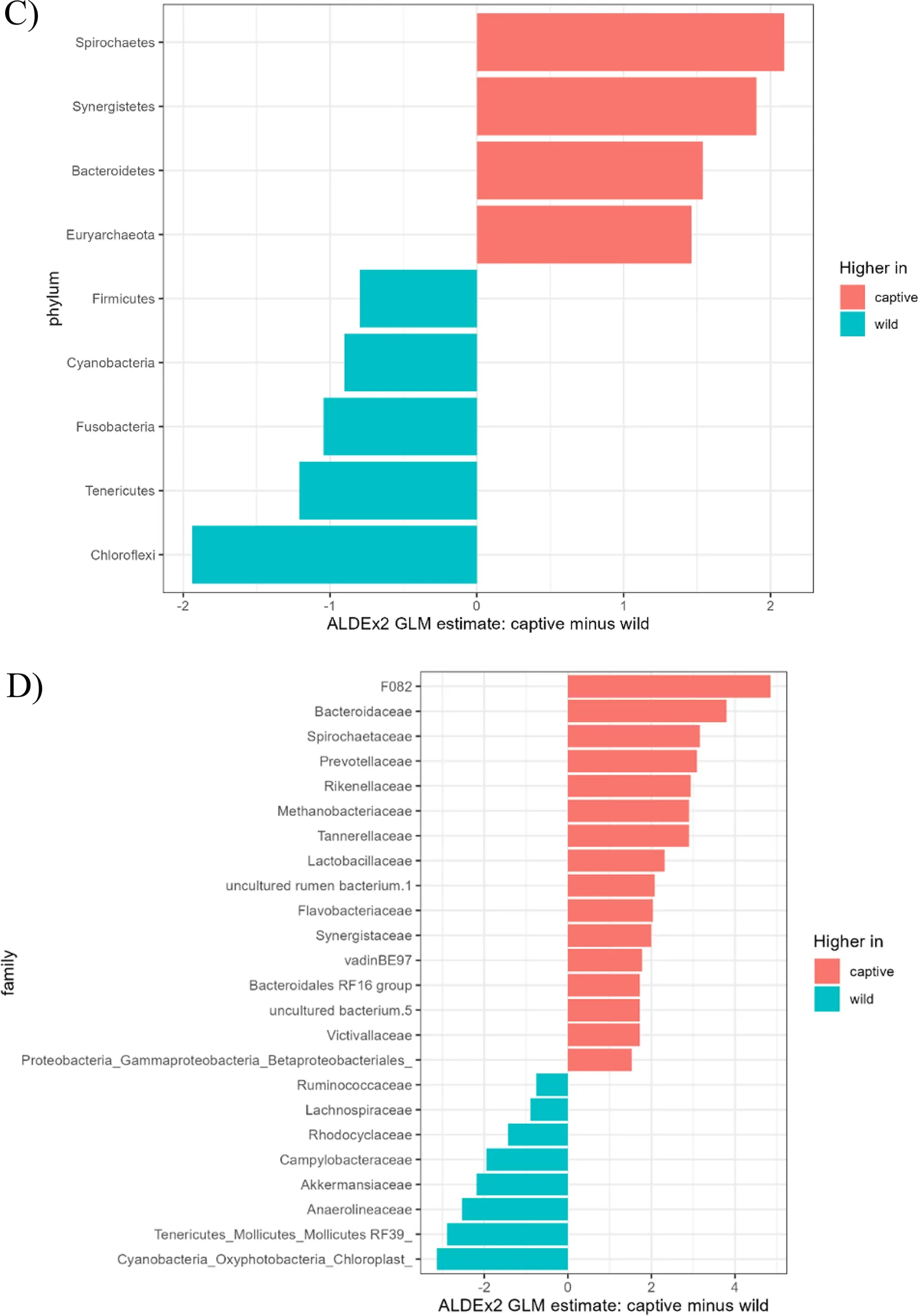
Effects of captivity on gut microbiome composition and diversity across primate taxa. (**A**) PCA of Aitchison distances calculated from centred log-ratio (CLR)-transformed family-level data comparing wild and captive individuals. (**B**) Observed Shannon diversity indices in wild and captive samples. Boxes represent interquartile ranges, centre lines indicate medians, and points represent individual samples. (**C**) ALDEx2 differential-abundance analysis at the phylum level comparing wild and captive individuals. Bars represent effect sizes; negative values indicate enrichment in wild individuals and positive values indicate enrichment in captive individuals. Only taxa passing Benjamini–Hochberg correction (BH-adjusted *p* < 0.05) are shown. (**D**) ALDEx2 differential-abundance analysis at the family level

**Fig. 5:**
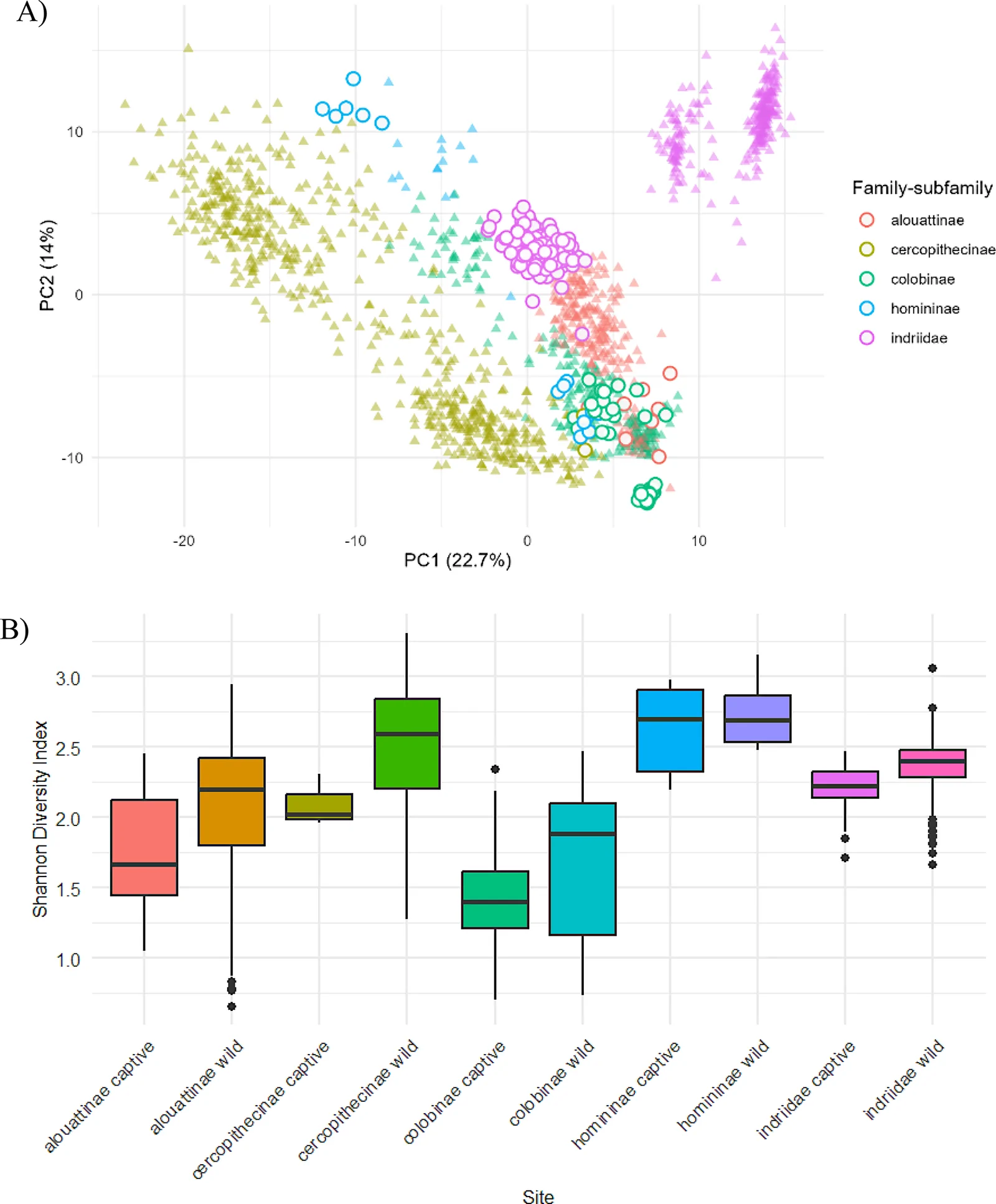
Family- and subfamily-level patterns of gut microbiome variation between wild and captive individuals. (**A**) PCA ordination of Aitchison distances from CLR-transformed abundance data, coloured by family–subfamily and shaped by site (wild vs. captive). Clustering reflects the combined effects of host phylogeny and captivity, with clear site-associated separation observed within the Indriidae family. (**B**) Shannon diversity indices across family–subfamily groups, stratified by site, showing consistently lower alpha diversity in captive individuals, with the largest reductions observed in Cercopithecinae and Colobinae

Observed Shannon diversity was lower in captive samples than in wild samples overall (wild mean = 2.25; captive mean = 2.06) (Fig. 4B) and within each family/subfamily (Fig. 5B). Consistent with this pattern, an unadjusted model including site alone estimated significantly lower diversity in captivity (estimate = − 0.198, F = 25.14, *p* = 5.93 × 10⁻⁷). To account for non-independence among studies, sequencing depth, and methodological variation, Shannon diversity was subsequently analysed using linear mixed-effects models that included site, family/subfamily, sequencing depth, sequencing instrument, sample stabilisation method, storage method, DNA extraction method, and study identity as a random effect. In this full methodological-covariate model, the estimated site effect was significant but reversed direction relative to the unadjusted model (site: F = 43.01, *p* = 7.42 × 10⁻¹¹), with significant variation among family/subfamily groups (site × family/subfamily: F = 2.90, *p* = 0.034). Adjusted captive diversity was higher in Alouattinae, Cercopithecinae, Colobinae and Homininae, while the Indriidae contrast was not estimable in the full model [see Additional file 17].

Diagnostic model comparisons showed that this reversal was driven primarily by adjustment for study identity rather than by sequencing depth, family/subfamily or methodological covariates alone [see Additional file 18]. Models including site plus family/subfamily retained the observed pattern of lower diversity in captivity (estimate = − 0.188, F = 40.10, *p* = 3.14 × 10⁻¹⁰), whereas models incorporating study identity reversed the estimated captivity effect (estimate = + 0.406, F = 64.08, *p* = 2.32 × 10⁻¹⁵).

The direction of the captivity effect was highly sensitive to study-level adjustment as several captive groups had very small sample sizes, and the full methodological model was partly rank-deficient due to confounding among study, site and technical variables; hence, the adjusted positive captivity effect must be interpreted cautiously. Together, these findings indicate substantial confounding between captivity status and study structure within the compiled dataset.

Beta diversity analyses based on Aitchison distance of CLR-transformed family-level data showed partial compositional separation between wild and captive primate microbiomes, although clustering patterns were more strongly structured by broader biological groupings such as family/subfamily and dietary strategy than by site alone (Fig. 4A; see Additional file 5 Fig. S3). In the adjusted PERMANOVA model including site and technical covariates as fixed effects, site explained a small but significant proportion of microbiome compositional variation (pseudo-F = 9.60, R² = 0.00223, *p* = 0.001). In contrast, technical variables, particularly storage method (pseudo-F = 106.49, R² = 0.04956, *p* = 0.001), accounted for a substantially larger proportion of variation, while stabilisation method also remained significant (pseudo-F = 35.95, R² = 0.01673, *p* = 0.001). Homogeneity of multivariate dispersion differed significantly between wild and captive samples (PERMDISP: F = 51.58, *p* = 0.001), indicating unequal within-group variability. Consequently, the observed PERMANOVA differences likely reflect both differences in centroid location and differences in dispersion between site conditions.

When site was analysed together with dietary strategy and fermentation type across all families and subfamilies, all three biological variables remained significant predictors of microbiome composition in the adjusted PERMANOVA model. Visualisation of the PCA ordination coloured by family/subfamily and site further showed that microbiome composition clustered primarily according to host taxonomic grouping, with several family/subfamily groups forming distinct clusters irrespective of captivity status (Fig. 5A). In contrast, wild and captive samples within the same family/subfamily generally showed partial overlap, although some groups, particularly Indriidae, displayed clearer separation between captive and wild samples. Fermentation strategy explained the largest proportion of variation among the biological variables (pseudo-F = 20.18, R² = 0.00459, *p* = 0.001), followed by diet (pseudo-F = 12.64, R² = 0.00288, *p* = 0.001) and site (pseudo-F = 10.39, R² = 0.00236, *p* = 0.001). Technical variables again contributed substantially to compositional variation, particularly storage method (pseudo-F = 106.10, R² = 0.04829, *p* = 0.001) and stabilisation method (pseudo-F = 30.26, R² = 0.01377, *p* = 0.001).

ALDEx2 fixed-effect GLM differential-abundance analysis identified significant wild-captive differences after adjustment for fermentation strategy, diet, sequencing instrument, stabilisation method, storage method and DNA extraction protocol, where estimable. At the phylum level, nine phyla differed significantly between wild and captive samples. Captive samples were enriched in Spirochaetes, Synergistetes, Bacteroidetes and Euryarchaeota, whereas wild samples were enriched in Chloroflexi, Tenericutes, Fusobacteria, Cyanobacteria and Firmicutes (BH-adjusted *p* < 0.05; Fig. 4C; see Additional file 8).

At the family level, 24 family-level features differed significantly between wild and captive samples in the adjusted GLM. Sixteen were enriched in captive samples, including F082, Bacteroidaceae, Spirochaetaceae, Prevotellaceae, Rikenellaceae, Methanobacteriaceae, Tannerellaceae, Lactobacillaceae, Flavobacteriaceae and Synergistaceae. Eight were enriched in wild samples, including Ruminococcaceae, Lachnospiraceae, Rhodocyclaceae, Campylobacteraceae, Akkermansiaceae, Anaerolineaceae, Mollicutes RF39 and a chloroplast-assigned cyanobacterial feature (BH-adjusted *p* < 0.05; Fig. 4D; see Additional file 9).

Sensitivity analysis using ALDEx2-CLR linear mixed models with study identity as a random effect supported the direction of the fixed-effect GLM results for taxa significant in the primary model, but identified a larger number of significant taxa overall [see Additional file 19 and 20]. Therefore, the adjusted fixed-effect ALDEx2 GLM was retained as the primary differential-abundance analysis, with mixed-model outputs provided as supplementary sensitivity analyses.

### Faecal microbiome core microbiome in wild colobines

**Fig. 6:**
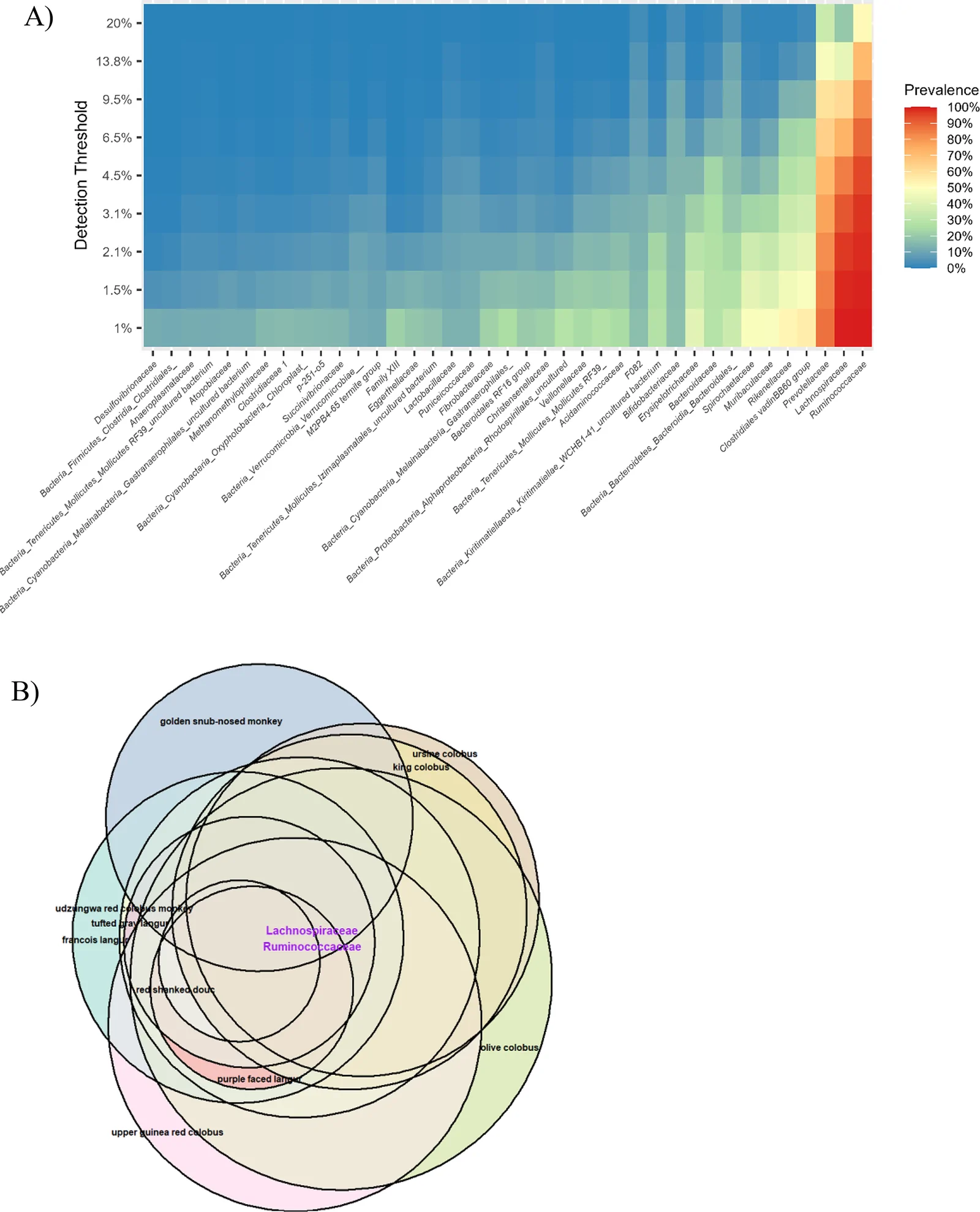
Core faecal microbiome composition of wild colobines. (**A**) Core microbiome heatmap showing the prevalence of bacterial families across a range of detection thresholds (minimum abundance). Colours indicate prevalence (percentage of samples in which a taxon is detected) for each detection threshold. (**B**) Venn diagram illustrating the overlap of core bacterial families among wild colobine species included in the meta-analysis. Ruminococcaceae and Lachnospiraceae were consistently identified as core members across species

The wild colobine species included in the meta-analysis were François langur (*Trachypithecus francoisi*), Udzungwa red colobus monkey (*Piliocolobus gordonorum*), Tufted gray langur (*Semnopithecus priam*), Purple-faced langur (*Semnopithecus vetulus*), Golden snub-nosed monkey (*Rhinopithecus roxellana*), Ursine colobus (*Colobus vellerosus*), Red-shanked douc (*Pygathrix nemaeus*), King colobus (*Colobus* polykomos), Olive colobus (*Procolobus verus*), and Upper Guinea red colobus (*Piliocolobus badius*). At the family level, Ruminococcaceae, Lachnospiraceae, Clostridiales vadinBB60 group, Christensenellaceae and Muribaculaceae were the most prevalent in the faecal core microbiome of wild colobines (Fig. 6A-B). Core microbiome per colobine species can be found in an additional file [see Additional file 10].

### Captivity is associated with altered microbiome composition in colobines

Given that colobines were the primary focus of this meta-analysis and displayed distinctive microbiome characteristics, including higher Firmicutes abundance and comparatively low alpha diversity, the effect of captivity on colobine faecal microbiome was examined separately.

**Fig. 7:**
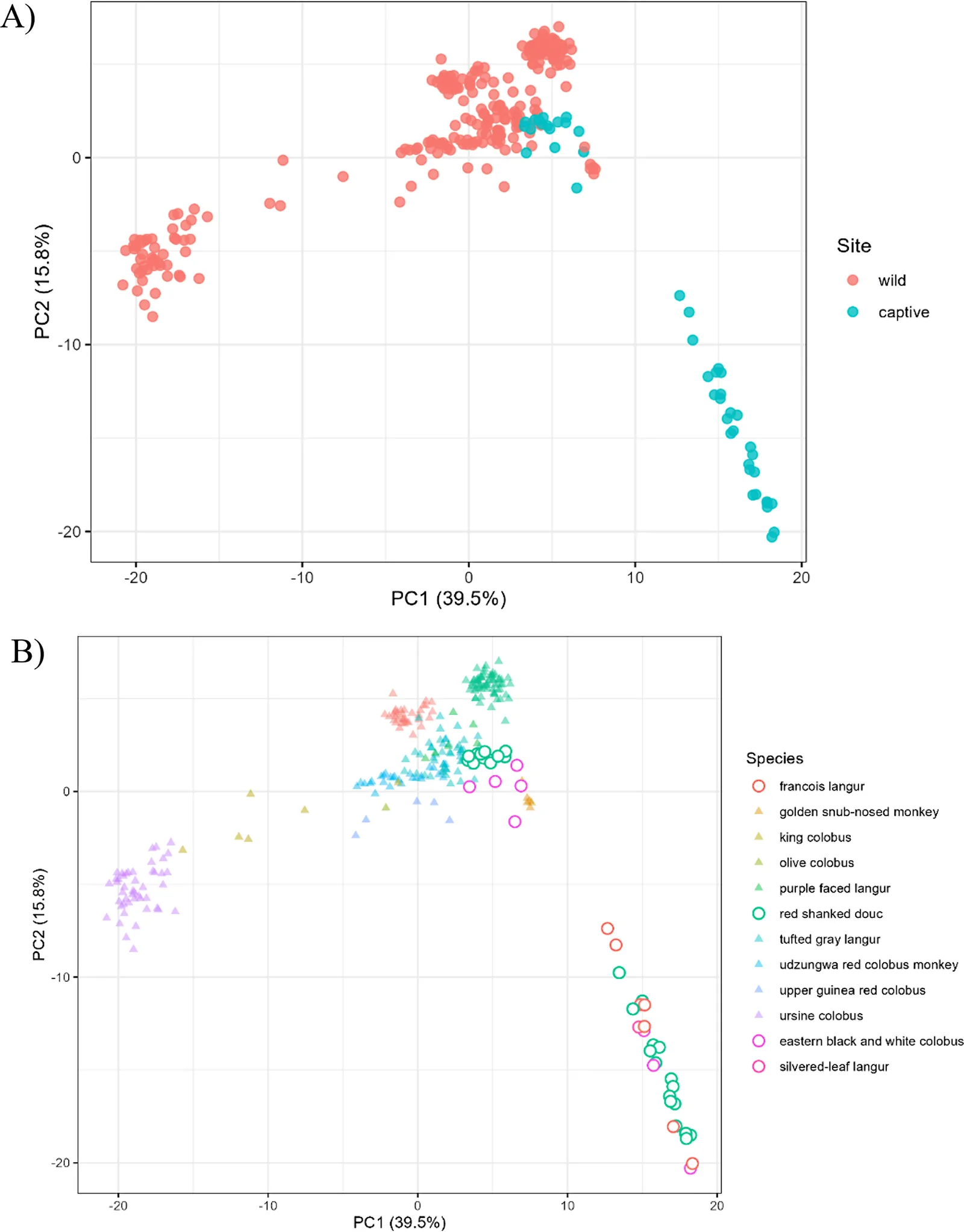

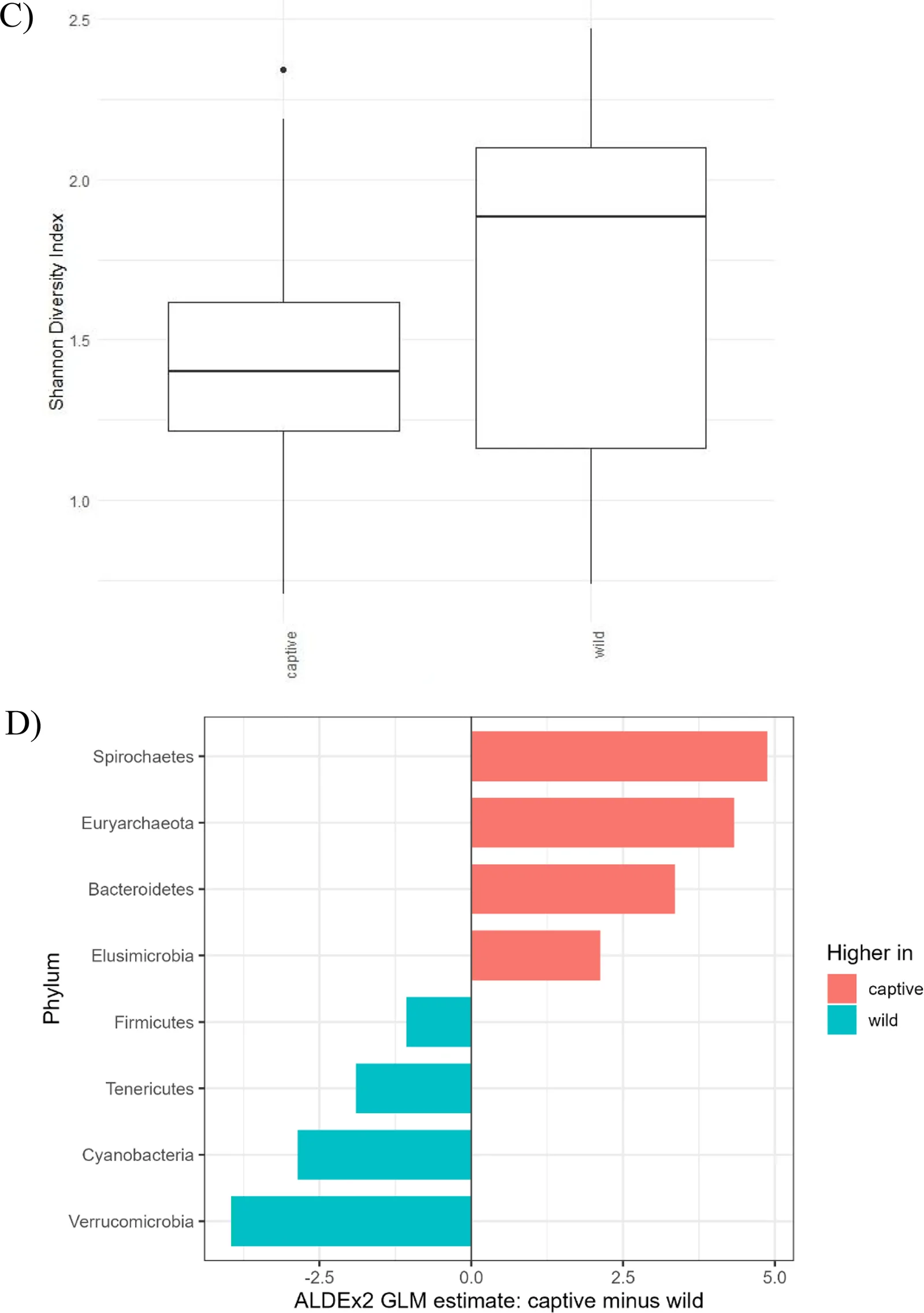

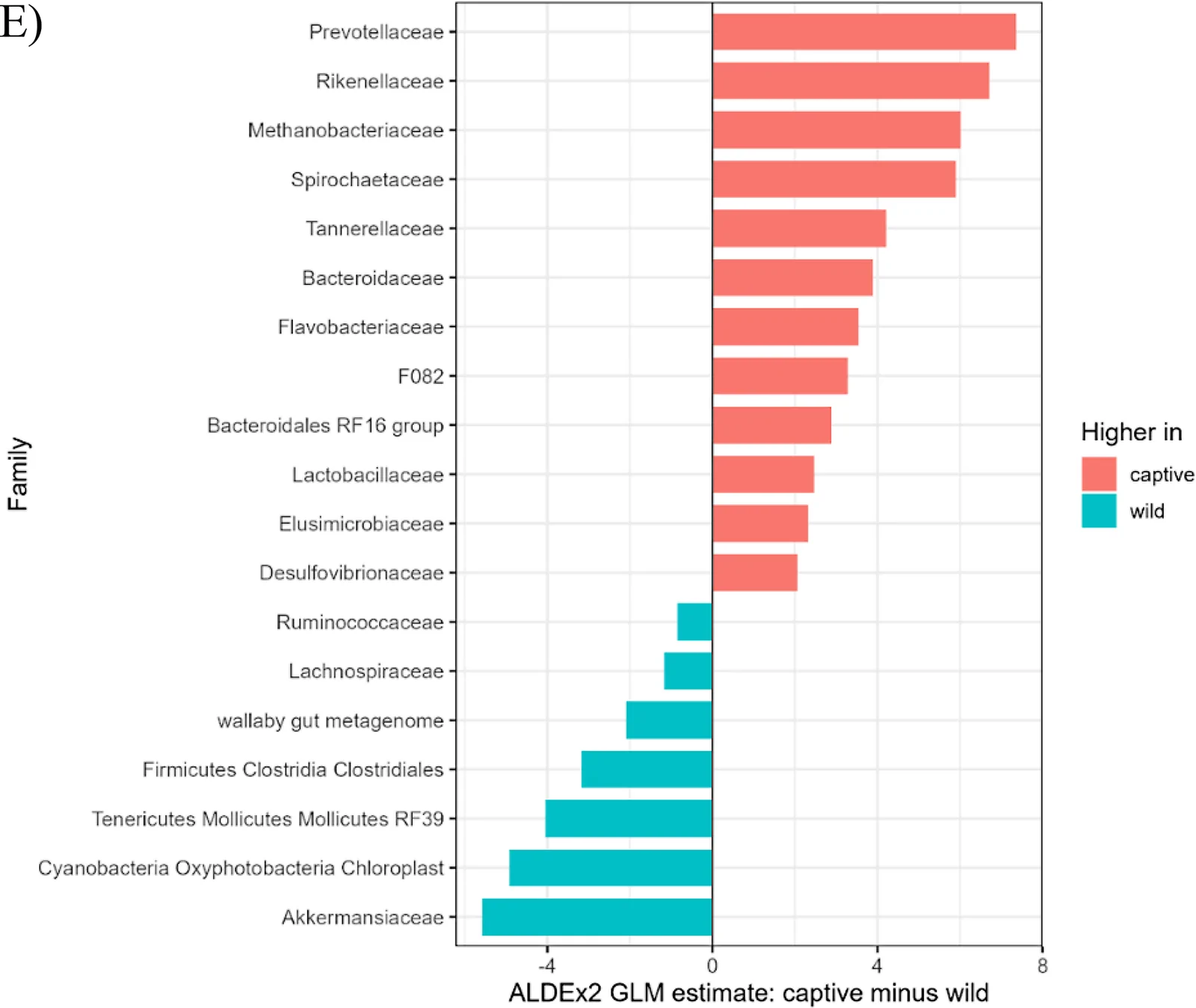
Captivity effects on the colobine faecal microbiome. (**A**) PCA of Aitchison distances calculated from centred log-ratio (CLR)-transformed family-level data comparing wild and captive colobine samples. (**B**) PCA of Aitchison distances calculated from centred log-ratio (CLR)-transformed family-level data coloured by colobine species and site. (**C**) Shannon diversity indices in wild and captive colobines. Boxes represent interquartile ranges, centre lines indicate medians, and points represent individual samples. (**D**) ALDEx2 differential-abundance analysis at the phylum level comparing wild and captive colobines. Negative effect sizes indicate enrichment in wild colobines and positive effect sizes indicate enrichment in captive colobines. Only taxa with BH-adjusted *p* < 0.05 are shown. (**E**) ALDEx2 differential-abundance analysis at the family level

Observed Shannon diversity was lower in captive colobines than in wild colobines (wild: *n* = 250, mean = 1.72, median = 1.88; captive: *n* = 45, mean = 1.43, median = 1.40; Fig. 7B). Consistent with this raw pattern, an unadjusted model including site alone estimated significantly lower Shannon diversity in captivity (estimate = − 0.282, F = 13.24, *p* = 3.24 × 10⁻⁴). At the species level, however, this pattern was not uniform among species represented in both wild and captive samples: captive François’ langurs had lower mean Shannon diversity than wild François’ langurs, whereas captive red-shanked doucs had higher mean Shannon diversity than wild red-shanked doucs [see Additional file 21].

Because all samples in this subset were colobines, foregut fermenters and folivores, family/subfamily, fermentation type and diet were not included as covariates. Shannon diversity was analysed using a linear mixed-effects model including site, log-transformed read depth, sequencing instrument, sample stabilisation method, storage method, DNA extraction method and study identity as a random effect. In this full methodological-covariate model, the estimated site effect reversed direction relative to the unadjusted model, with captive colobines showing higher adjusted Shannon diversity than wild colobines (estimate = + 0.549; site: F = 80.61, *p* < 2.2 × 10⁻¹⁶). Log-transformed read depth was also a significant predictor of Shannon diversity (F = 27.37, *p* = 3.27 × 10⁻⁷), whereas sequencing instrument, stabilisation method and storage method were not significant predictors. DNA extraction method was not independently estimable in the full model [see Additional file 22].

Diagnostic model comparisons showed that the reversal was not simply caused by sequencing depth. The unadjusted site-only model retained the observed pattern of lower diversity in captivity (estimate = − 0.282, F = 13.24, *p* = 3.24 × 10⁻⁴), whereas adding species identity reversed the estimated site effect (estimate = + 0.152, F = 9.54, *p* = 0.0022). Models incorporating study identity also estimated higher adjusted diversity in captivity, both without species identity (estimate = + 0.482, F = 59.11, *p* = 2.27 × 10⁻¹³) and with sequencing depth included (estimate = + 0.535, F = 77.24, *p* = 1.34 × 10⁻¹⁶) [see Additional file 23].

This adjusted result should be interpreted cautiously because captivity status was strongly confounded with study identity, technical variables and species representation. The full methodological model was rank deficient, with several storage and DNA-extraction terms dropped, and adjusted marginal means for wild and captive groups were not estimable, although the captive-*versus*-wild contrast was estimable. Thus, although the raw data show lower alpha diversity in captive colobines, the adjusted models indicate that this pattern is highly sensitive to species composition, study structure and methodological covariates.

Beta diversity analyses based on Aitchison distance of CLR-transformed family-level data showed partial compositional separation between wild and captive colobine samples (Fig. 7A; see Additional file 5 Fig. S4). In the adjusted PERMANOVA model, site explained a significant but modest proportion of microbiome compositional variation (pseudo-F = 18.91, R² = 0.01941, *p* = 0.001). In contrast to broader primate analyses, homogeneity of multivariate dispersion did not differ significantly between wild and captive colobines (PERMDISP: F = 0.072, *p* = 0.796), indicating that the observed PERMANOVA differences primarily reflected differences in centroid location rather than unequal within-group variability. Visualisation of the PCA ordination further showed that microbiome composition clustered strongly according to colobine species, with several species forming distinct groups irrespective of captivity status (Fig. 7B; see Additional file 5 Fig. S5). Wild and captive samples from the same species generally showed partial overlap, although some captive individuals occupied more distinct positions in ordination space. PC1 explained 39.5% of the variation and PC2 explained 15.8%.

ALDEx2 fixed-effect GLM differential abundance analysis identified significant wild-captive differences in colobine microbiome composition at both phylum and family levels after adjustment for sequencing instrument, stabilisation method, storage method and DNA extraction protocol, where estimable. Because all samples in this subset were colobines, foregut fermenters and folivores, family/subfamily, fermentation type and diet were not included as covariates [Additional files 11 and 12].

At the phylum level, eight phyla differed significantly between wild and captive colobines. Captive colobines were enriched in Spirochaetes, Euryarchaeota, Bacteroidetes and Elusimicrobia, whereas wild colobines were enriched in Verrucomicrobia, Cyanobacteria, Tenericutes and Firmicutes (BH-adjusted *p* < 0.05; Fig. 7C).

At the family level, 19 family-level features differed significantly between wild and captive colobines. Wild colobines were enriched in Ruminococcaceae, Lachnospiraceae, Akkermansiaceae, Mollicutes RF39 and other Firmicutes/Clostridiales-associated features. In contrast, captive colobines were enriched in Prevotellaceae, Rikenellaceae, Methanobacteriaceae, Spirochaetaceae, Tannerellaceae, Bacteroidaceae, Flavobacteriaceae, Elusimicrobiaceae, Lactobacillaceae and Desulfovibrionaceae (BH-adjusted *p* < 0.05; Fig. 7D).

ALDEx2-CLR mixed-model sensitivity analyses, including study identity as a random effect, supported the direction of the primary fixed-effect GLM results, and full mixed-model outputs are provided as supplementary sensitivity analyses [Additional file 24 and 25].

## Discussion

In this meta-analysis, we compared colobine species with other non-foregut-fermenting herbivorous primate species, including folivores and non-folivores, to explore factors contributing to susceptibility to gastrointestinal health issues of colobines in captivity. Our results revealed systematic differences in the faecal microbiome of colobine primates compared to those of other primate families, which are associated with their specialised folivorous diet and foregut fermentation strategy.

Across wild primate families and subfamilies, both alpha and beta diversity differed significantly, indicating clear baseline distinctions in gut microbial composition. Firmicutes was the dominant phylum in most families/subfamilies, whereas Bacteroidetes predominated in Indriidae and Lemuridae. Among all groups, colobines exhibited the most distinctive microbiome profile, characterised by the highest relative abundance of Firmicutes and the lowest relative abundance of Bacteroidetes in wild populations, together with the lowest alpha diversity.

The low diversity observed in wild colobines likely reflects a highly specialised microbial community co-adapted to foregut fermentation and the efficient breakdown of fibrous plant material. However, such specialisation may come at a cost: a reduced pool of functionally redundant taxa, which could make these communities less resilient to perturbations such as dietary change or environmental stress [5, 51, 52]. Thus, the distinctive but low-diversity microbiome of colobines may underpin both their efficient fibre digestion and their heightened vulnerability to gastrointestinal imbalance under altered conditions. These results are broadly consistent with previous studies reporting Firmicutes enrichment and reduced microbial diversity in wild colobines across habitats and species [7, 13, 15, 18], supporting the view that their gut microbiome represents a uniquely specialised and potentially fragile ecosystem.

Unlike all other primate families, which rely on hindgut fermentation, colobines are the only primates to have evolved foregut fermentation within a multi-chambered stomach. Our comparison between foregut-fermenting colobines and hindgut-fermenting taxa, therefore, captured the influence of fermentation physiology on microbial community composition. The results revealed significant differences in both alpha and beta diversity between fermentation strategies: foregut fermenters exhibited significantly lower alpha diversity and distinct compositional profiles. Differential-abundance analyses identified enrichment of Firmicutes, and Verrucomicrobia in foregut fermenters, together with enrichment of several fibre-associated bacterial families, including Ruminococcaceae, Lachnospiraceae, Akkermansiaceae, Peptococcaceae and Christensenellaceae. Many of these taxa are associated with fibre degradation, short-chain fatty acid production, and mucin metabolism [53–55], consistent with the heavy reliance of colobines on microbial fermentation for energy extraction.

Similar microbial signatures have been described in ruminant foregut communities, and a previous study showed that gut microbiota clustered by fermentation physiology rather than host phylogeny [56]. A direct comparison of colobines, non-colobine primates, and wild ruminants likewise found that colobine faecal communities shared many of the same fibre-degrading taxa found in ruminant foregut, indicating similarity driven by digestive strategy. However, the proportional representation of these taxa more closely resembled other non-colobine primates, suggesting that functional guilds are conserved, whereas relative abundance remained closer to the primate profile. These parallels suggest that the colobine gut microbiome represents an intermediate state between those of ruminants and other primates, highly specialised for fibre fermentation but potentially limited in functional redundancy [57].

However, these differences may not be explained solely by fermentation physiology, as colobines also possess an exceptionally selective folivorous diet, which could further shape their gut microbial community. To disentangle the influence of diet from fermentation physiology, we next compared folivorous and non-folivorous primate families. This analysis extended beyond colobines to include other folivores, such as howlers (Alouattinae) and folivorous lemurs (Indriidae and Lepilemuridae), thereby addressing a broader dietary question. The results revealed significant differences in both alpha and beta diversity: folivores showed consistently lower alpha diversity than non-folivores, and beta-diversity analyses demonstrated significant compositional differences between dietary groups.

Differential-abundance analyses further showed enrichment of Firmicutes, Fibrobacteres and Verrucomicrobia in folivores, together with enrichment of several fibre-associated bacterial families, including Lachnospiraceae and Ruminococcaceae. These findings align with previous comparative studies showing that folivorous primates generally exhibit microbial communities specialised for structural carbohydrate fermentation [7, 58]. Importantly, fibre-associated taxa were enriched in folivores across both foregut and hindgut fermenting species, suggesting that adaptation to high-fibre diets may favour convergent microbial functions irrespective of fermentation site.

The colobine gut microbiome may be inherently less diverse than that of other primates, reflecting strong functional specialisation for fibre fermentation. While such specialisation likely enhances digestive efficiency under natural dietary conditions, it may also come at the cost of reduced microbial redundancy and resilience. In ecological systems, lower species diversity often corresponds to decreased stability and adaptability to perturbations [59, 60], and similar principles have been applied to microbial ecosystems [27, 28]. In humans, lower gut microbial diversity has been associated with a range of chronic inflammatory and metabolic conditions, including inflammatory bowel disease, obesity, and type 2 diabetes [61–63]. However, the relationship between diversity and health is complex, and there is no consensus that lower diversity is synonymous with gastrointestinal illness. Consequently, we cannot assume a causal association between low diversity and disease. For example, Amato et al. [1] found no significant diversity differences between healthy and gastrointestinally compromised red-shanked douc langurs (*Pygathrix nemaeus*). Emerging evidence indicates that diversity may influence health indirectly, through effects on community stability and functional redundancy, rather than via diversity per se [64]. Consequently, future studies should incorporate functional and compositional analyses (e.g., metagenomics, metabolomics) alongside diversity metrics. It is therefore likely that the low diversity of colobine microbiomes does not indicate that they are inherently “unhealthy,” but rather that they are finely tuned to a narrow ecological niche. Under natural conditions, this may represent a stable equilibrium; in captivity, however, deviations in diet, stress, or habitat may push this system beyond its resilience threshold. Thus, the combination of naturally low diversity and high specialisation may contribute to the apparent vulnerability of colobines to gastrointestinal dysregulation in captivity.

When comparing wild and captive individuals across all primate families and subfamilies, both alpha and beta diversity differed significantly between environments. However, ordination analyses indicated that microbiome clustering was more strongly structured by broader biological grouping, including host family/subfamily, dietary strategy, and fermentation type, than by captivity status alone. Several family/subfamily groups formed distinct clusters irrespective of site, while wild and captive individuals within the same family/subfamily groups often showed partial overlap. Interestingly, Indriidae showed one of the clearest separations between wild and captive samples in the ordination analyses. However, the causes of this pattern remain uncertain because captive and wild samples were derived from a limited number of studies with differing environmental and husbandry conditions. Nevertheless, captivity status remained a significant predictor of microbiome composition after adjustment for technical covariates. Although the proportion of explained variance attributable to site was relatively small, these results indicate that captivity consistently influences microbial community structure across herbivorous primates. Captivity-associated shifts were particularly apparent in several folivorous groups, including Colobinae and, to a lesser extent, Alouattinae, both of which rely heavily on microbial fermentation.

Differential abundance analyses using ALDEx2 revealed significant shifts in several bacterial taxa under captive conditions, including Bacteroidetes, Spirochaetes, and Euryarchaeota at the phylum level, and Prevotellaceae, Rikenellaceae, Bacteroidaceae, Tannerellaceae, Methanobacteriaceae, and Spirochaetaceae at the family level. In contrast, wild individuals retained higher relative abundance of fibre-associated taxa including Ruminococcaceae, Lachnospiraceae and Akkermansiaceae. Overall, these findings align with previous research demonstrating that captivity induces compositional convergence among captive individuals and enrichment of human-associated bacterial taxa in non-human primates [24, 65]. The patterns observed here suggest that captivity imposes selective pressures that partially shift the gut microbiome away from its natural, fibre-adapted configuration, affecting folivorous and foregut-fermenting species such as colobines.

Importantly, despite significant captivity-associated shifts, host biology remained the dominant determinant of microbiome structure across the dataset. This likely explains why the effects of captivity were statistically detectable but accounted for only a relatively small proportion of the overall compositional variance. Fermentation strategy, dietary category, and host family/subfamily consistently explained stronger clustering patterns than captivity status alone. These findings highlight the strong evolutionary and ecological constraints shaping gut microbial communities in herbivorous primates while suggesting that captivity-related changes occur within this broader biological framework.

Focusing specifically on colobines, captivity was associated with significant changes in gut microbial composition, while alpha-diversity patterns required cautious interpretation because of study-level and methodological confounding. Wild and captive colobines differed significantly in beta diversity, although ordination analyses still showed partial overlap between groups, indicating that core aspects of the colobine microbiome remain conserved across environments. Captive colobines exhibited compositional shifts away from several key fibre-associated bacterial groups. At the phylum level, captive colobines were enriched in Spirochaetes, Euryarchaeota, Bacteroidetes, and Elusimicrobia, whereas wild colobines showed higher relative abundance of Verrucomicrobia, Cyanobacteria, Tenericutes, and Firmicutes. At the family level, wild colobines retained higher abundance of Ruminococcaceae, Lachnospiraceae, Akkermansiaceae, and Mollicutes RF39, whereas captive colobines were enriched in Prevotellaceae, Rikenellaceae, Spirochaetaceae, Methanobacteriaceae, Bacteroidaceae and Desulfovibrionaceae. Notably, Lachnospiraceae and Ruminococcaceae were identified as key components of the wild colobine core microbiome in our study. Both groups are well known for their roles in plant-fibre degradation and short-chain fatty acid (SCFA) production, especially butyrate, which supports gut-barrier integrity and anti-inflammatory function [53, 54]. Akkermansiaceae, particularly *Akkermansia spp.*, has also been associated with mucin turnover and gut barrier maintenance [55]. Lower relative abundance of these taxa under captive conditions may therefore reflect a shift away from the specialised fibre-fermenting configuration observed in wild colobines. Captive colobines were also enriched in Desulfovibrionaceae, a family of sulfate-reducing bacteria that can produce hydrogen sulfide and has been associated with inflammatory gut conditions in humans, among other non-gastrointestinal diseases [66].

Several factors could contribute to the changes seen in colobine microbiomes in captivity, including altered diet composition (reduced fibre and increased starch or fruit sugars), limited microbial exposure, antibiotic and disinfectant use, or chronic stress [65, 67–69]. Moreover, the impact of captivity likely varies depending on the specific context, such as whether individuals are long-term zoo residents born in captivity or wild-caught animals temporarily or permanently housed in rescue centres following injury or illness. Animals transferred from the wild may experience abrupt dietary and environmental transitions or pre-existing health issues, both of which could further exacerbate microbial instability. Together, these findings indicate that captivity, under a range of management circumstances, disrupts the finely balanced microbial ecosystem that characterises wild colobines. Recent colobine studies, published subsequent to the time frame covered by our meta-analysis, collectively support our findings, reporting that captivity tends to reduce microbial diversity [26] and shifts communities away from fibre-degrading Firmicutes toward Bacteroidetes/Prevotellaceae [21, 22]. Although these taxa are not necessarily pathogenic, their enrichment may indicate restructuring of the gut microbiome away from the highly specialised fermentative configuration observed in wild folivorous colobines.

The findings from this meta-analysis underscore the importance of considering the unique gut microbiome of colobines, shaped by their folivorous diet and foregut-fermenting physiology, as a central determinant of their health and well-being in captivity. The pronounced reduction in core fibre-degrading taxa, such as Ruminococcaceae and Lachnospiraceae, suggests that current captive diets may be poorly matched to the nutritional ecology of these primates. Diets should therefore be carefully reformulated to replicate the fibre composition, plant diversity, and secondary metabolite profile of wild diets as closely as possible [4]. Such dietary adjustments are likely to enhance fermentative efficiency and restore a more balanced microbial community.

In addition to dietary optimisation, proactive microbiome management could help maintain gut health in captivity. Routine monitoring of key microbial indicators, particularly the abundance of key members of Ruminococcaceae and Lachnospiraceae, may serve as an early warning system for dysbiosis, allowing timely dietary or veterinary interventions. This approach could be applied to both adult colobines and infants, whose developing gut microbiota may be especially sensitive to environmental and nutritional perturbations.

For established gastrointestinal imbalance, therapeutic strategies such as targeted probiotics or faecal microbiota transplantation (FMT) may be valuable adjuncts. Probiotics designed to promote colonisation by beneficial, fibre-degrading bacteria could support digestion and nutrient absorption in captive settings. FMT from healthy individuals has shown promise in restoring microbial diversity and function in other mammals [70, 71]. However, further research using shotgun metagenomics and longitudinal studies will be essential to identify the core bacterial species and metabolic pathways critical for maintaining colobine gut homeostasis, and to guide evidence-based microbiome therapies.

A major limitation of this meta-analysis is the considerable methodological heterogeneity among the source studies. Sampling, storage, DNA extraction, sequencing platforms, and analytical pipelines all varied widely, and such differences are known to influence microbiome profiles and diversity estimates [72, 73]. Technical variables, particularly storage method, also explained a substantial proportion of microbiome variation across the combined dataset. These effects were therefore explicitly accounted for in multivariable analyses together with sequencing instrument, stabilisation method, and DNA extraction protocol where estimable. Nevertheless, residual methodological heterogeneity remains an inherent limitation of large-scale cross-study microbiome meta-analyses and should be considered when interpreting fine-scale taxonomic differences.

These results highlight the critical need for methodological standardisation in primate microbiome research. Future meta-analyses should aim to include as many datasets as possible to balance potential biases introduced by individual studies and ensure adequate representation of each methodological category. Ultimately, establishing common standards for sample preservation, DNA extraction, and sequencing protocols will greatly enhance the comparability and reproducibility of microbiome research, allowing more accurate assessments of biological drivers such as diet, physiology, and captivity.

Several additional limitations should also be acknowledged. First, despite the inclusion of 16 studies and 1,690 samples, some primate families and captive groups remained represented by relatively small sample sizes, particularly within colobines. Second, the included datasets differed in sequencing platforms, sample preservation methods, extraction protocols, and 16 S rRNA variable regions, potentially introducing residual technical variation despite statistical adjustment. And third, faecal samples provide only an indirect representation of microbial processes occurring within different gut compartments, particularly in foregut-fermenting species where microbial activity is concentrated in the forestomach.

## Conclusions

This study, despite limitations, provides the largest comparative analysis to date of the gut microbiome of herbivorous primates, with particular focus on the unique microbial ecology of colobines. Our findings demonstrate that colobines possess a highly specialised microbiome strongly associated with folivory and foregut fermentation, characterised by a high abundance of Firmicutes and enrichment of fibre-associated families such as Ruminococcaceae and Lachnospiraceae. Captivity was associated with significant restructuring of this microbial system, particularly through compositional shifts away from the wild fibre-adapted microbiome configuration. These results provide an important framework for future studies investigating the functional consequences of captivity-associated microbiome shifts and may help inform husbandry and dietary management strategies for one of the most challenging primate groups to maintain successfully in captivity.

Although observed alpha diversity was generally lower in captive individuals, adjusted alpha-diversity models indicated that these patterns were strongly influenced by study identity, species representation, sequencing depth, and methodological variables. Consequently, alpha-diversity differences should be interpreted cautiously rather than as a simple unconfounded effect of captivity. The strongest evidence for captivity-associated microbiome disruption came from consistent compositional differences detected by beta-diversity and differential abundance analyses.

Historical challenges with gastrointestinal health in captive settings, coupled with our current findings, suggest that colobines may be particularly susceptible to microbiome disruption under ex-situ conditions. Comparative studies between colobines and other wild foregut fermenters could provide valuable insight into the relative contributions of digestive physiology and diet to gut microbiome evolution. Future research should prioritise longitudinal studies spanning wild-to-captive transitions, controlled dietary interventions, and functional approaches such as metagenomics and metabolomics to determine how captivity-associated shifts in the microbiome affect digestive function and health. Such work could inform evidence-based dietary and microbiome management strategies with important implications for colobine welfare, ex-situ conservation, and reintroduction programmes.

## Supplementary Information

Below is the link to the electronic supplementary material.

Supplementary Material 1

Supplementary Material 2

Supplementary Material 3

Supplementary Material 4

Supplementary Material 5

Supplementary Material 6

Supplementary Material 7

Supplementary Material 8

Supplementary Material 9

Supplementary Material 10

Supplementary Material 11

Supplementary Material 12

Supplementary Material 13

Supplementary Material 14

Supplementary Material 15

Supplementary Material 16

Supplementary Material 17

Supplementary Material 18

Supplementary Material 19

Supplementary Material 20

Supplementary Material 21

Supplementary Material 22

Supplementary Material 23

Supplementary Material 24

Supplementary Material 25

## Data Availability

The sequencing datasets analysed in this study were obtained from previously published studies and are available in the original publications and their associated public repositories (ENA, SRA, Qiita, and EBI), which are included in the Additional file 1. In addition, some datasets were provided directly by the original authors and are not publicly available; these data were used with permission and cannot be redistributed. Requests for access to these datasets should be directed to the original data owners.The R script used for data processing and statistical analyses is provided in the Additional file 16.
