# Supplementary material for: Colobine gut microbiome vulnerability to captivity: a meta-analysis of herbivorous primates
Source: Anim Microbiome. 2026 Jul 29;8:110. doi: 10.1186/s42523-026-00600-6 (PMC13715106; doi:10.1186/s42523-026-00600-6)
Supplement: Supplementary file 1 [file 42523_2026_600_MOESM16_ESM.pdf]

```
#Load packages

library(phyloseq)

library(microbiome)

library(ggplot2)

library(vegan)

library(dplyr)

library(openxlsx)

library(tidyverse)

library(zCompositions)

library(ecodist)

library(writexl)


#CREATE PHYLOSEQ OBJECT


#Get metadata and remove unnecessary samples/information

metadata <- read.csv("Metadata1.csv", row.names=1, header=TRUE, strip.white = TRUE, sep=",")

#Only keep feces samples, healthy animals and subset of families

meta_sub <- subset(metadata, gi.sample.site == "feces" & gi.health != "unhealthy" &
family.subfamily %in% c("colobinae","cercopithecinae", "homininae", "alouattinae", "indriidae",
"lemuridae", "lepilemuridae") & site == "wild")

meta_sub <- subset(metadata, gi.sample.site == "feces" & gi.health != "unhealthy" &
family.subfamily %in% c("colobinae","cercopithecinae", "homininae", "alouattinae", "indriidae",
"lemuridae", "lepilemuridae"))

meta_sub <- subset(metadata, gi.sample.site == "feces" & gi.health != "unhealthy" &
family.subfamily == "colobinae" & site == "wild")

#Remove metadata table from memory

rm("metadata")


#Load OTU table

otu_table <- read.table("otu_table.txt",sep="\t",row.names = 1, header=TRUE, check.names =
FALSE)

#Force to matrix

otu_table <- as.matrix(otu_table)
```

```
#Select those samples which are in the subset of the meta data

otu_table <- otu_table[,row.names(meta_sub)]

#Remove OTUs with zero counts (table contains zero counts as result of sample removal):

otu_table <- otu_table[which(rowSums(otu_table) > 0), ]

#Check column and row numbers

ncol(otu_table)#1458/199 Should give same number as meta_sub observations

nrow(otu_table)#78328/19882


#Column sum gives "mapped reads" per sample, explore range across all samples:

csums <- colSums(otu_table)

min(csums)#1969/716

max(csums)#510839/136968


#Row sums gives counts per OTU, explore range across all OTUs:

rsums <- rowSums(otu_table)

min(rsums)#1/1

max(rsums)#696971/338938


# Load taxonomy #

taxonomy <- read.csv("taxonomy1.csv",sep=",",row.names=1, header=TRUE)

taxonomy <- as.matrix(taxonomy)

#Only select those OTU IDs which are in the OTU table after subsetting

taxonomy <- taxonomy[row.names(otu_table),]

head(taxonomy)#See how this looks

nrow(taxonomy)# 78328/19882 Should be same as OTU table


# Process inputs to phyloseq object #

OTU <- otu_table(otu_table, taxa_are_rows= TRUE)

TAX <- tax_table(taxonomy, errorIfNULL = TRUE)

META <- sample_data(meta_sub)
```

```
# Create object

pseq<- phyloseq(OTU, TAX, META)

pseq


#FILTER PHYLOSEQ OBJECT


# remove singletons and doubletons #

#singletons - OTU only appears once (=have one read mapped)

#Doubeltons - OTU only appears twice (=have only two reads mapped in one or more samples)

#Code modified because function from filtering script led to complete read removal for many samples!

pseq_otu <- filter_taxa(pseq, function (x) {sum(x) > 3}, prune=TRUE)

#check lowest read numbers

summarize_phyloseq(pseq_otu)


# Remove ambiguous taxa - taxa not classified at high levels #

pseq_filt <- subset_taxa(pseq_otu, !is.na(Phylum) & !Phylum %in% c("", "uncharacterized", "Ambiguous_taxa"))## remove undefined phyla


# check how samples and otus after general filtering

summarize_phyloseq(pseq_filt) #Looks ok, no singletons


#Aggregate taxa to higher taxonomic levels.

pseq_family <- aggregate_taxa(pseq_filt, "Family")

pseq_genus <- aggregate_taxa(pseq_filt, "Genus")

rank_names(pseq_family)

rank_names(pseq_genus)

# Rename 'unique' to 'Genus'

colnames(tax_table(pseq_family))[colnames(tax_table(pseq_family)) == "unique"] <- "Genus"

colnames(tax_table(pseq_genus))[colnames(tax_table(pseq_genus)) == "unique"] <- "Genus"

#Have a look at all the Families in the dataset:

family_table <- table(tax_table(pseq_family)[, "Family"])
```

```
# Convert to data frame

family_df <- as.data.frame(family_table)

colnames(family_df) <- c("Family", "Count")

# Save to Excel

library(writexl)

write_xlsx(family_df, "family_counts_family.xlsx")


#Here I make a list of Families which I want to remove (e.g. protozoa, fungi, and plant names),

#these taxa are mainly in there because of chloroplast and mitochondria hits, German cockroach seems to refer to a bacterium,

#thus it is left in (in doubt, check full taxonomic path in taxonomy1.csv file):

to_be_removed <- c("Ammopiptanthus mongolicus", "Ambiguous_taxa", "Anemone flaccida", "Eimeria praecox",

        "Euglena deses", "Mitochondria", "Millettia pinnata", "Pardosa pseudoannulata",

        "Quercus robur", "Solanum pennellii", "Tanaecium tetragonolobum", "Phytophthora lateralis MPF4",

        "Virgulinella fragilis", "Vismia ferruginea", "Ziziphus jujuba", "Bryum argenteum var. argenteum")


#Remove taxa

pseq_family_filt <- subset_taxa(pseq_family, !is.na(Family) & !Family %in% to_be_removed)

summarize_phyloseq(pseq_family_filt)

pseq_genus_filt <- subset_taxa(pseq_genus, !is.na(Family) & !Family %in% to_be_removed)


#Convert to relative abundance

fam_rel_abund <- microbiome::transform(pseq_family_filt, "compositional")

phyloseq::otu_table(pseq_family_filt)[1:5, 1:5]


gen_rel_abund <- microbiome::transform(pseq_genus_filt, "compositional")

phyloseq::otu_table(pseq_genus_filt)[1:5, 1:5]


#Create Custom Color Palette
```

```
library(RColorBrewer)

# Create a color palette with 10 distinct colors
my_colors <- brewer.pal(n = 35, name = "Set1")# set1 only 9 colours

#Make plot for data exploration on Phylum level, group data by "family.subfamily"
p <- phyloseq::plot_bar(fam_rel_abund, fill = "Phylum") +
  geom_bar(aes(color = Phylum, fill = Phylum), stat = "identity", position = "stack") +
  labs(x = "", y = "Relative Abundance\n") +
  facet_wrap(~ study.name, scales = "free") +
  theme(panel.background = element_blank(),
        axis.text.x=element_blank(),
        axis.ticks.x=element_blank(),
        text = element_text(size = 8))  # Adjust the size as needed
p
# Save the plot
ggsave("dataexploration_study.png", plot = p, width = 8, height = 6, dpi = 300)

#Agglomerate to phylum-level and rename
ps_phylum <- phyloseq::tax_glom(fam_rel_abund, "Phylum", NArm=FALSE)
phyloseq::taxa_names(ps_phylum) <- phyloseq::tax_table(ps_phylum)[, "Phylum"]
phyloseq::otu_table(ps_phylum)[1:5, 1:5]
#Melt and plot family
phyloseq::psmelt(ps_phylum) %>%
  ggplot(data = ., aes(x = diet, y = Abundance)) +
  geom_boxplot(outlier.shape  = NA) +
  geom_jitter(aes(color = Phylum), height = 0, width = .2) +
  labs(x = "", y = "Relative Abundance\n") +
  facet_wrap(~ Phylum, scales = "free") +
  theme(axis.text.x = element_text(angle = 90, hjust = 1))
```

```
##Subset by phylum but before we need to make the table to know the most important phylum

####Table %

# Assuming fam_rel_abund is a phyloseq object, we need to extract the abundance and metadata tables

otu1 <- abundances(ps_phylum)

meta1 <- meta(fam_rel_abund)


# Transpose the OTU table to get samples as rows and taxa as columns (if needed)

otu1 <- t(otu1)

# Convert relative abundances to percentages

otu_percentages <- otu1 * 100


# Ensure the OTU table has rownames as the same order of samples as in metadata

# Assuming the metadata dataframe has a 'family.subfamily' column for grouping

otu_meta1 <- data.frame(otu_percentages, study.name = meta1$study.name)

otu_meta2 <- data.frame(otu_percentages, instrument = meta1$instrument)

otu_meta3 <- data.frame(otu_percentages, stabilization.method = meta1$stabilization.method)

otu_meta4 <- data.frame(otu_percentages, storage = meta1$storage)

otu_meta5 <- data.frame(otu_percentages, dna.extraction = meta1$dna.extraction)

# Assuming otu_meta1 contains OTU table with percentages and 'family.subfamily' column

# Convert the data to long format for easier manipulation

otu_long <- otu_meta1 %>%

  pivot_longer(cols = -study.name, names_to = "Phylum", values_to = "Abundance")


otu_long2 <- otu_meta2 %>%

  pivot_longer(cols = -instrument, names_to = "Phylum", values_to = "Abundance")


otu_long3 <- otu_meta3 %>%

  pivot_longer(cols = -stabilization.method, names_to = "Phylum", values_to = "Abundance")


otu_long4 <- otu_meta4 %>%
```

```
  pivot_longer(cols = -storage, names_to = "Phylum", values_to = "Abundance")

otu_long5 <- otu_meta5 %>%
  pivot_longer(cols = -dna.extraction, names_to = "Phylum", values_to = "Abundance")

# Group the data by 'family.subfamily' and 'Phylum', and calculate the mean abundance
phylum_summary <- otu_long %>%
  group_by(study.name, Phylum) %>%
  summarise(Mean_Abundance = mean(Abundance), .groups = "drop")

phylum_summary2 <- otu_long2 %>%
  group_by(instrument, Phylum) %>%
  summarise(Mean_Abundance = mean(Abundance), .groups = "drop")

phylum_summary3 <- otu_long3 %>%
  group_by(stabilization.method, Phylum) %>%
  summarise(Mean_Abundance = mean(Abundance), .groups = "drop")

phylum_summary4 <- otu_long4 %>%
  group_by(storage, Phylum) %>%
  summarise(Mean_Abundance = mean(Abundance), .groups = "drop")

phylum_summary5 <- otu_long5 %>%
  group_by(dna.extraction, Phylum) %>%
  summarise(Mean_Abundance = mean(Abundance), .groups = "drop")

# Print the summary
print(phylum_summary)
print(phylum_summary2)
print(phylum_summary3)
print(phylum_summary4)
```

```
print(phylum_summary5)

# Write the relative abundance table to an Excel file
write_xlsx(phylum_summary, path = "relative_abundance_allphylum_study.xlsx")
write_xlsx(phylum_summary2, path = "relative_abundance_allphylum_instrument.xlsx")
write_xlsx(phylum_summary3, path = "relative_abundance_allphylum_stabilization.xlsx")
write_xlsx(phylum_summary4, path = "relative_abundance_allphylum_storage.xlsx")
write_xlsx(phylum_summary5, path = "relative_abundance_allphylum_dna-extraction.xlsx")

#statisitcs to compare mean abundance

###Select and change phylum to include from the table you just made
# Melt and filter the data to include only the selected phyla
selected_phyla <- c("Actinobacteria", "Bacteroidetes", "Chloroflexi", "Cyanobacteria",
"Euryarchaeota", "Fibrobacteres", "Firmicutes","Kiritimatiellaeota", "Planctomycetes",
"Proteobacteria", "Spirochaetes","Tenericutes", "Verrucomicrobia")

melted_data <- phyloseq::psmelt(ps_phylum) # Adjust this line accor

# Filter for the selected phyla
filtered_data <- melted_data %>%
  filter(Phylum %in% selected_phyla)  # Adjust this line according to the actual column name for
phyla in your data

# Plot the data with boxplot on top of jittered sample dots
p <- ggplot(filtered_data, aes(x = study.name, y = Abundance)) +
  geom_jitter(aes(color = OTU), height = 0, width = .2) +  # Draw jittered points first
  geom_boxplot(outlier.shape = NA) +  # Draw the boxplot on top
  labs(x = "", y = "Relative Abundance\n") +
  facet_wrap(~ OTU, scales = "free", ncol = 5) +  # Arrange 5 plots per row
  theme(axis.text.x = element_text(angle = 90, hjust = 1))
p
```

```
p1 <- p + theme(
  axis.text.x = element_text(angle = 45, vjust = 1, hjust = 1, size = 4), # Adjust x-axis text size
  axis.text.y = element_text(size = 6), # Adjust y-axis text size
  legend.text = element_text(size = 6), # Adjust legend text size
  legend.title = element_text(size = 8), # Adjust legend title size
  plot.margin = margin(t = 30, r = 30, b = 30, l = 40) # Adjust margins
)
p1

# Save the plot
ggsave("selectedphyla_dna.png", plot = p1, width = 8, height = 6, dpi = 300)

# for reproducibility
set.seed(90210)

#ALPHA DIVERSITY
alpha_tab <-microbiome::alpha(fam_rel_abund, index = "shannon")
#Look at estimated diversity metrics:
head(alpha_tab)
#Statistical testing of alpha diversity
#Construct the data table
dtable <- meta(fam_rel_abund)
dtable$diversity <- alpha_tab$diversity_shannon
#Write data to file for future easy access
write.csv(dtable, file = "shannon_data_folivore.csv", row.names = FALSE)
#Look at distribution of response variable (Shannon's diversity)
hist(dtable$diversity)

#Perform Statistical Comparison alpha
#Use ANOVA or Kruskal-Wallis test to compare Shannon diversity between family.subfamily groups.
```

##to check if data meets anova assumptions

library(car)

#1. Independence of Observations

#This assumption is usually checked based on the study design. If the design ensures that the samples are independent, this assumption is considered to be met.

#2. Normality of Residuals

#You can check the normality of residuals using a QQ plot and the Shapiro-Wilk test.

Fit the ANOVA model

anova_model1 <- aov(diversity ~ study.name, data = dtable)

anova_model2 <- aov(diversity ~ instrument, data = dtable)

anova_model3 <- aov(diversity ~ stabilization.method, data = dtable)

anova_model4 <- aov(diversity ~ storage, data = dtable)

anova_model5 <- aov(diversity ~ dna.extraction, data = dtable)

anova_model6 <- aov(diversity ~ Family.Site, data = dtable)

Extract the residuals

residuals1 <- residuals(anova_model1)

residuals2 <- residuals(anova_model2)

residuals3 <- residuals(anova_model3)

residuals4 <- residuals(anova_model4)

residuals5 <- residuals(anova_model5)

residuals6 <- residuals(anova_model6)

QQ plot to visually check normality

qqnorm(residuals1)

qqline(residuals1, col = "red")

qqnorm(residuals2)

qqline(residuals2, col = "red")

qqnorm(residuals3)

qqline(residuals3, col = "red")

```
qqnorm(residuals4)
qqline(residuals4, col = "red")


qqnorm(residuals5)
qqline(residuals5, col = "red")


qqnorm(residuals6)
qqline(residuals6, col = "red")
# Shapiro-Wilk test for normality
shapiro_test1 <- shapiro.test(residuals1)
print(shapiro_test1)
shapiro_test2 <- shapiro.test(residuals2)
print(shapiro_test2)
shapiro_test3 <- shapiro.test(residuals3)
print(shapiro_test3)
shapiro_test4 <- shapiro.test(residuals4)
print(shapiro_test4)
shapiro_test5 <- shapiro.test(residuals5)
print(shapiro_test5)
shapiro_test6 <- shapiro.test(residuals6)
print(shapiro_test6)
# Check homogeneity of variances
# Convert family.subfamily to a factor
dtable$study.name <- as.factor(dtable$study.name)
dtable$instrument <- as.factor(dtable$instrument)
dtable$stabilization.method <- as.factor(dtable$stabilization.method)
dtable$storage <- as.factor(dtable$storage)
dtable$dna.extraction <- as.factor(dtable$dna.extraction)
dtable$Family.Site <- as.factor(dtable$Family.Site)
```

```
dtable$family.subfamily <- as.factor(dtable$family.subfamily)

dtable$fermentation <- as.factor(dtable$fermentation)

dtable$diet.category <- as.factor(dtable$diet.category)

# Perform Levene's test for homogeneity of variances

library(car)

levene_test1 <- leveneTest(diversity ~ study.name, data = dtable)

print(levene_test1)


levene_test2 <- leveneTest(diversity ~ instrument, data = dtable)

print(levene_test2)


levene_test3 <- leveneTest(diversity ~ stabilization.method, data = dtable)

print(levene_test3)


levene_test4 <- leveneTest(diversity ~ storage, data = dtable)

print(levene_test4)


levene_test5 <- leveneTest(diversity ~ dna.extraction, data = dtable)

print(levene_test5)


levene_test6 <- leveneTest(diversity ~ Family.Site, data = dtable)

print(levene_test6)


#Interpretation

#QQ Plot: Look at the QQ plot to see if the points fall along the line. If they do, it suggests normality.

#Shapiro-Wilk Test: The p-value should be greater than 0.05 to support the assumption of normality.

#Levene's Test: The p-value should be greater than 0.05 to support the assumption of equal variances.

#If either of the Shapiro-Wilk or Levene's test has a p-value less than 0.05, consider using the Kruskal-Wallis test instead of ANOVA.
```

```
# If assumptions met -->Perform ANOVA to compare Shannon diversity between family.subfamily groups

anova_result <- aov(diversity ~ family.subfamily, data = dtable)

summary(anova_result)


# Alternatively, if data does not meet ANOVA assumptions, use Kruskal-Wallis test

#The Kruskal-Wallis test is a non-parametric method for testing whether samples originate from the same distribution. It is an appropriate alternative when the assumptions of ANOVA are not met.

kruskal_result1 <- kruskal.test(diversity ~ analysis, data = dtable)

print(kruskal_result1)


kruskal_result2 <- kruskal.test(diversity ~ instrument, data = dtable)

print(kruskal_result2)


kruskal_result3 <- kruskal.test(diversity ~ stabilization.method, data = dtable)

print(kruskal_result3)


kruskal_result4 <- kruskal.test(diversity ~ storage, data = dtable)

print(kruskal_result4)


kruskal_result5 <- kruskal.test(diversity ~ dna.extraction, data = dtable)

print(kruskal_result5)


kruskal_result6 <- kruskal.test(diversity ~ Family.Site, data = dtable)

print(kruskal_result6)


# Visualize the results with a boxplot

ggplot(dtable, aes(x = Family.Site, y = diversity, fill = Family.Site)) +

 geom_boxplot() +

 theme_minimal() +
```

```
 labs(
  title = "Shannon Diversity by Site and Family-Subfamily Group",
  x = "Site",
  y = "Shannon Diversity Index"
 ) +
 theme(
  axis.text.x = element_text(angle = 45, hjust = 1),
  legend.position = "none"  # Removes the legend
 )

#plot

ggplot(dtable, aes(x = Family.Site, y = diversity, fill = Family.Site)) +
 geom_boxplot() +
 theme_minimal() +
 labs(
  title = "Shannon Diversity by Site and Family-Subfamily Group",
  x = "Site",
  y = "Shannon Diversity Index"
 ) +
 theme(
  axis.text.x = element_text(angle = 45, hjust = 1, size = 8),  # x-axis labels
  axis.text.y = element_text(size = 8),                    # y-axis labels
  axis.title.x = element_text(size = 8),                   # x-axis title
  axis.title.y = element_text(size = 8),                   # y-axis title
  plot.title = element_text(size = 12, face = "bold"),         # plot title
  legend.position = "none"                              # remove legend
 )

#plot wild and captive same colour
dtable_wica <- dtable %>%
```

```
  mutate(SiteType = ifelse(grepl("captive", Family.Site), "Captive", "Wild"))

#ggplot(dtable, aes(x = Family.Site, y = diversity, fill = SiteType)) +
ggplot(dtable_wica, aes(x = Family.Site, y = diversity, fill = SiteType)) +
  geom_boxplot() +
  theme_minimal() +
  labs(
    title = "Shannon Diversity by Site and Family-Subfamily Group",
    x = "Site",
    y = "Shannon Diversity Index"
  ) +
  theme(
    axis.text.x = element_text(angle = 45, hjust = 1, size = 8),
    axis.text.y = element_text(size = 8),
    axis.title.x = element_text(size = 8),
    axis.title.y = element_text(size = 8),
    plot.title = element_text(size = 12, face = "bold")
  ) +
  scale_fill_manual(values = c("Captive" = "#d95f02", "Wild" = "#1b9e77"))

##same but with gaps between families
#Create a custom order of Family.Site including "gap" levels:
dtable_wica$Family.Site <- factor(dtable_wica$Family.Site, levels = c(
  "alouattinae wild", "alouattinae captive",
  "gap1",
  "cercopithecinae wild", "cercopithecinae captive",
  "gap2",
  "colobinae wild", "colobinae captive",
  "gap3",
  "homininae wild", "homininae captive",
  "gap4",
```

```
  "indriidae wild", "indriidae captive"
))

#Then add rows with NA for diversity to the dataset for the gaps:
# Create a gap row for each gap level, with same structure as dtable_wica
gaps <- dtable_wica[0, ]  # empty dataframe with same structure
gaps <- rbind(
  gaps,
  data.frame(Family.Site = factor("gap1", levels = levels(dtable_wica$Family.Site))),
  data.frame(Family.Site = factor("gap2", levels = levels(dtable_wica$Family.Site))),
  data.frame(Family.Site = factor("gap3", levels = levels(dtable_wica$Family.Site))),
  data.frame(Family.Site = factor("gap4", levels = levels(dtable_wica$Family.Site)))
)

# Fill in missing columns with NA
for (col in names(dtable_wica)) {
  if (!col %in% names(gaps)) {
    gaps[[col]] <- NA
  }
}

# Reorder columns to match
gaps <- gaps[, names(dtable_wica)]

# Now bind them
dtable_with_gaps <- rbind(dtable_wica, gaps)

#Define x-axis labels with gaps blanked
gap_labels <- c(
  "alouattinae wild" = "alouattinae\nwild",
  "alouattinae captive" = "alouattinae\ncaptive",
```

```
  "gap1" = "",
  "cercopithecinae wild" = "cercopithecinae\nwild",
  "cercopithecinae captive" = "cercopithecinae\ncaptive",
  "gap2" = "",
  "colobinae wild" = "colobinae\nwild",
  "colobinae captive" = "colobinae\ncaptive",
  "gap3" = "",
  "homininae wild" = "homininae\nwild",
  "homininae captive" = "homininae\ncaptive",
  "gap4" = "",
  "indriidae wild" = "indriidae\nwild",
  "indriidae captive" = "indriidae\ncaptive"
)


#plot
ggplot(dtable_with_gaps, aes(x = Family.Site, y = diversity, fill = SiteType)) +
  geom_boxplot(na.rm = TRUE) +
  scale_x_discrete(labels = gap_labels) +
  theme_minimal() +
  labs(
    title = "Shannon Diversity by Site and Family-Subfamily Group",
    x = "Site",
    y = "Shannon Diversity Index"
  ) +
  theme(
    axis.text.x = element_text(angle = 45, hjust = 1, size = 8),
    axis.text.y = element_text(size = 8),
    axis.title.x = element_text(size = 8),
    axis.title.y = element_text(size = 8),
    plot.title = element_text(size = 12, face = "bold")
```

```
 ) +
 scale_fill_manual(values = c("Captive" = "#d95f02", "Wild" = "#1b9e77"), na.translate = FALSE)



##facet by family
#extract Family
dtable_wica <- dtable_wica %>%
 mutate(Family = sub(" (captive|wild)$", "", Family.Site))
#plot facet
ggplot(dtable_wica, aes(x = SiteType, y = diversity, fill = SiteType)) +
 geom_boxplot() +
 facet_wrap(~ Family, scales = "free_x") +
 theme_minimal() +
 labs(
  title = "Shannon Diversity by Site Type and Primate Family",
  x = "Site Type",
  y = "Shannon Diversity Index"
 ) +
 theme(
  strip.text = element_text(face = "bold", size = 10),
  axis.text.x = element_text(size = 8),
  axis.text.y = element_text(size = 8),
  axis.title.x = element_text(size = 8),
  axis.title.y = element_text(size = 8),
  plot.title = element_text(size = 12, face = "bold")
 ) +
 scale_fill_manual(values = c("Wild" = "#1b9e77", "Captive" = "#d95f02"))



# compare shannon index by site and family
```

```
library(dplyr)

diversity_summary <- dtable %>%
  group_by(family.subfamily, site) %>%
  summarize(mean_diversity = mean(diversity, na.rm = TRUE), .groups = "drop") %>%
  pivot_wider(names_from = site, values_from = mean_diversity)

diversity_summary <- diversity_summary %>%
  mutate(abs_change = abs(`wild` - `captive`))

biggest_change <- diversity_summary %>%
  arrange(desc(abs_change)) %>%
  slice(1)

print(biggest_change)

write.csv(diversity_summary, "diversity_summary.csv", row.names = FALSE)

#
ggplot(dtable, aes(x = family.subfamily, y = diversity, fill = family.subfamily)) +
  geom_boxplot() +
  theme_minimal() +
  labs(
    title = "Shannon Diversity by Family-Subfamily Group",
    x = "Family-Subfamily",
    y = "Shannon Diversity Index"
  ) +
  theme(axis.text.x = element_text(angle = 90, hjust = 1))

# Perform pairwise comparisons using Wilcoxon rank-sum test
```

pairwise_results1 <- pairwise.wilcox.test(dtable$diversity, dtable$family.subfamily, p.adjust.method = "BH")

print(pairwise_results1)

pairwise_results2 <- pairwise.wilcox.test(dtable$diversity, dtable$instrument, p.adjust.method = "BH")

print(pairwise_results2)

pairwise_results3 <- pairwise.wilcox.test(dtable$diversity, dtable$stabilization.method, p.adjust.method = "BH")

print(pairwise_results3)

pairwise_results4 <- pairwise.wilcox.test(dtable$diversity, dtable$storage, p.adjust.method = "BH")

print(pairwise_results4)

pairwise_results5 <- pairwise.wilcox.test(dtable$diversity, dtable$dna.extraction, p.adjust.method = "BH")

print(pairwise_results5)

####After Alpha, we need to do clr transformation to continue workflow (distance Aitchinson, ordination PCA, multivariate comparison permanova o anosim, correlation)

#Add a pseudocount and perform the CLR transformation:

Assuming pseq_family_filt is your phyloseq object

Extract the count data and add pseudocount

otu_counts <- otu_table(pseq_family_filt)

otu_counts_pseudo <- otu_counts + 1

Update the OTU table with the pseudocount-added data

otu_table(pseq_family_filt) <- otu_table(otu_counts_pseudo, taxa_are_rows = taxa_are_rows(otu_counts))

```
# Perform CLR transformation
clr_transformed <- microbiome::transform(pseq_family_filt, "clr")



##Calculate Aitchison distance


# Extract the OTU table from the phyloseq object
clr_phylum_assay <- otu_table(clr_transformed)


# Transposes it to get taxa to columns
clr_phylum_assay <- t(clr_phylum_assay)


# Calculates Euclidean distances between samples. Because taxa is in columns,
# it is used to compare different samples.
euclidean_phylum_dist <- vegan::vegdist(clr_phylum_assay, method = "euclidean")


# Print the distances
print(euclidean_phylum_dist)


##to export to excel convert to data frame
# Convert the dist object to a matrix
ait_matrix <- as.matrix(euclidean_phylum_dist)


# Convert the matrix to a data frame
ait_df <- as.data.frame(ait_matrix)


#to excel
write_xlsx(ait_df, path = "aitchisondistances.xlsx")
##


# Does principal coordinate analysis
```

```
euclidean_phylum_pcoa <- ecodist::pco(euclidean_phylum_dist)

# Creates a data frame from principal coordinates
euclidean_phylum_pcoa_df <- data.frame(
 pcoa1 = euclidean_phylum_pcoa$vectors[,1],
 pcoa2 = euclidean_phylum_pcoa$vectors[,2])

# Creates a plot
euclidean_phylum_plot <- ggplot(data = euclidean_phylum_pcoa_df,
                   aes(x=pcoa1, y=pcoa2)) +
 geom_point() +
 labs(x = "PC1",
    y = "PC2",
    title = "Aitchison distances at Family level") +
 theme(title = element_text(size = 12)) # makes titles smaller

euclidean_phylum_plot

##permanova from aitchison
#Get meta data from phyloseq object
meta2 <- meta(clr_transformed)

# Perform PERMANOVA..use data matrix version of aitchison distance
permanova_result1 <- adonis2(ait_matrix ~ study.name, data = meta2)
print(permanova_result1)

permanova_result <- adonis2(ait_matrix ~ analysis, data = meta2)
print(permanova_result)

permanova_result2 <- adonis2(ait_matrix ~ instrument, data = meta2)
print(permanova_result2)
```

```
permanova_result3 <- adonis2(ait_matrix ~ stabilization.method, data = meta2)
print(permanova_result3)

permanova_result4 <- adonis2(ait_matrix ~ storage, data = meta2)
print(permanova_result4)

permanova_result5 <- adonis2(ait_matrix ~ dna.extraction, data = meta2)
print(permanova_result5)

# Principal Coordinate Analysis (previously done- euclidean_phylum_pcoa)

# Creates a data frame from principal coordinates
pcoa_df <- data.frame(
 pcoa1 = euclidean_phylum_pcoa$vectors[,1],
 pcoa2 = euclidean_phylum_pcoa$vectors[,2],
 group = meta2$analysis
)



pcoa_df1 <- data.frame(
 pcoa1 = euclidean_phylum_pcoa$vectors[,1],
 pcoa2 = euclidean_phylum_pcoa$vectors[,2],
 group1 = meta2$study.name
)

pcoa_df2 <- data.frame(
 pcoa1 = euclidean_phylum_pcoa$vectors[,1],
 pcoa2 = euclidean_phylum_pcoa$vectors[,2],
 group2 = meta2$instrument
)
```

```
pcoa_df3 <- data.frame(
  pcoa1 = euclidean_phylum_pcoa$vectors[,1],
  pcoa2 = euclidean_phylum_pcoa$vectors[,2],
  group3 = meta2$stabilization.method
)

pcoa_df4 <- data.frame(
  pcoa1 = euclidean_phylum_pcoa$vectors[,1],
  pcoa2 = euclidean_phylum_pcoa$vectors[,2],
  group4 = meta2$storage
)

pcoa_df5 <- data.frame(
  pcoa1 = euclidean_phylum_pcoa$vectors[,1],
  pcoa2 = euclidean_phylum_pcoa$vectors[,2],
  group5 = meta2$dna.extraction
)
# Creates a plot
PCA_plot <- ggplot(data = pcoa_df, aes(x = pcoa1, y = pcoa2, color = group)) +
  geom_point(size = 3) +
  labs(
    x = "PC1",
    y = "PC2",
    title = "Aitchison distances by Analysis",
    color = "analysis") +
  theme_minimal() +
  theme(
    title = element_text(size = 12),
    legend.title = element_text(size = 10),
    legend.text = element_text(size = 8)
```

```
 )
PCA_plot


PCA_plot1 <- ggplot(data = pcoa_df1, aes(x = pcoa1, y = pcoa2, color = group1)) +
 geom_point(size = 3) +
 labs(
  x = "PC1",
  y = "PC2",
  title = "Aitchison distances by Study",
  color = "study.name") +
 theme_minimal() +
 theme(
  title = element_text(size = 12),
  legend.title = element_text(size = 10),
  legend.text = element_text(size = 8)
 )
PCA_plot1

PCA_plot2 <- ggplot(data = pcoa_df2, aes(x = pcoa1, y = pcoa2, color = group2)) +
 geom_point(size = 3) +
 labs(
  x = "PC1",
  y = "PC2",
  title = "Aitchison distances by Instrument",
  color = "Instrument") +
 theme_minimal() +
 theme(
  title = element_text(size = 12),
  legend.title = element_text(size = 10),
```

```
   legend.text = element_text(size = 8)
  )
PCA_plot2

PCA_plot3 <- ggplot(data = pcoa_df3, aes(x = pcoa1, y = pcoa2, color = group3)) +
  geom_point(size = 3) +
  labs(
   x = "PC1",
   y = "PC2",
   title = "Aitchison distances by Stabilization Method",
   color = "stabilization.method") +
  theme_minimal() +
  theme(
   title = element_text(size = 12),
   legend.title = element_text(size = 10),
   legend.text = element_text(size = 8)
  )
PCA_plot3

PCA_plot4 <- ggplot(data = pcoa_df4, aes(x = pcoa1, y = pcoa2, color = group4)) +
  geom_point(size = 3) +
  labs(
   x = "PC1",
   y = "PC2",
   title = "Aitchison distances by Storage",
   color = "storage") +
  theme_minimal() +
  theme(
   title = element_text(size = 12),
   legend.title = element_text(size = 10),
   legend.text = element_text(size = 8)
```

```
 )
PCA_plot4

PCA_plot5 <- ggplot(data = pcoa_df5, aes(x = pcoa1, y = pcoa2, color = group5)) +
 geom_point(size = 3) +
 labs(
  x = "PC1",
  y = "PC2",
  title = "Aitchison distances by DNA extraction method",
  color = "dna.extraction") +
 theme_minimal() +
 theme(
  title = element_text(size = 12),
  legend.title = element_text(size = 10),
  legend.text = element_text(size = 8)
 )
PCA_plot5

#ordination plot 2 variables

pcoa_df_two <- data.frame(
 pcoa1 = euclidean_phylum_pcoa$vectors[, 1],
 pcoa2 = euclidean_phylum_pcoa$vectors[, 2],
 family.subfamily = meta2$family.subfamily,
 site = meta2$site  # Add species.name here
)

unique_study_names <- unique(pcoa_df_two$family.subfamily)  # Check unique values
shape_values <- c(1, 2, 3, 4, 5, 6, 7, 8, 9, 10, 11, 12, 13, 14, 15, 16)  # Extend as needed
```

```
PCA_plot_two <- ggplot(data = pcoa_df_two, aes(x = pcoa1, y = pcoa2, color = family.subfamily,
shape = site)) +
 geom_point(size = 2) +
 labs(
  x = "PC1",
  y = "PC2",
  title = "Aitchison Distances by Site and Family-Subfamily",
  color = "Family-Subfamily",
  shape = "Site"
 ) +
 scale_shape_manual(values = shape_values) +  # Specify shapes manually
 theme_minimal() +
 theme(
  title = element_text(size = 6),
  legend.title = element_text(size = 5),
  legend.text = element_text(size = 5)
 )



PCA_plot_two <- PCA_plot_two +
 theme(
  plot.margin = margin(t = 14, r = 10, b = 14, l = 10)  # Adjust the margins (top, right, bottom, left)
 )


PCA_plot_two


#permanova for two variables
adonis_result <- adonis2(ait_matrix ~ family.subfamily + site, data = meta2, permutations = 999)
print(adonis_result)
```

#test assumptions

#Permutational Multivariate Analysis of Variance (PERMANOVA) is a non-parametric multivariate statistical test used to determine if there are significant differences between groups of samples based on a multivariate response variable. Before conducting PERMANOVA, it's important to ensure that the assumptions of the test are met.

#The main assumptions of PERMANOVA include:

1.Homogeneity of multivariate dispersions: This assumption is similar to the homogeneity of variances assumption in ANOVA, but in a multivariate context. It means that the spread or dispersion of data points within each group should be similar across all groups.

#Independence of observations: Each observation or sample should be independent of others. This means that there should be no systematic bias or correlation between observations.

```
# Assuming dist is your distance matrix and family.subfamily is your grouping variable

dispersion = betadisper(euclidean_phylum_dist, meta2$analysis)

anova(dispersion)


dispersion1 = betadisper(euclidean_phylum_dist, meta2$study.name)

anova(dispersion1)


dispersion2 = betadisper(euclidean_phylum_dist, meta2$instrument)

anova(dispersion2)


dispersion3 = betadisper(euclidean_phylum_dist, meta2$stabilization.method)

anova(dispersion3)


dispersion4 = betadisper(euclidean_phylum_dist, meta2$storage)

anova(dispersion4)


dispersion5 = betadisper(euclidean_phylum_dist, meta2$dna.extraction)

anova(dispersion5)


dispersion5 = betadisper(euclidean_phylum_dist, meta2$dna.extraction)
```

anova(dispersion5)

#2.Inspect Results and Residuals:

#Check the results of the betadisper test. A significant result suggests that the assumption of homogeneity of dispersion is violated.

plot(dispersion1)

plot(dispersion)

plot(dispersion2)

plot(dispersion3)

plot(dispersion4)

plot(dispersion5)

#If homogeneity violated, alternatives:

If dispersion is significant, consider transformations or alternative analyses

Perform PERMDISP

permdisp_result <- permutest(dispersion, permutations = 999)

print(permdisp_result)

permdisp_result1 <- permutest(dispersion1, permutations = 999)

print(permdisp_result1)

permdisp_result2 <- permutest(dispersion2, permutations = 999)

print(permdisp_result2)

permdisp_result3 <- permutest(dispersion3, permutations = 999)

print(permdisp_result3)

permdisp_result4 <- permutest(dispersion4, permutations = 999)

print(permdisp_result4)

```
permdisp_result5 <- permutest(dispersion5, permutations = 999)

print(permdisp_result5)


#DIFFERENTIAL ABUDANCE ALDEX2

library(ALDEx2)

#

sample_data <- data.frame(sample_data(pseq_family_filt))

count_data <- as.matrix(otu_table(pseq_family_filt))


# Subset data for specific pairwise comparison

site_subset <- subset(sample_data, site %in% c("wild", "captive"))

count_data_subset <- count_data[, colnames(count_data) %in% rownames(site_subset)]


# Perform ALDEx2 analysis on subset

ald_result <- aldex(count_data_subset, site_subset$site, denom="all", mc.samples=295)


str(ald_result)


#Verify Taxa Names

taxa_names <- rownames(count_data)

#Check ald_result Structure:

str(ald_result)

#Align Taxa Names:

taxa_names_subset <- taxa_names[1:nrow(ald_result)]

#Add Taxa Names to ald_result

ald_result$Taxon <- taxa_names_subset

#export o excel

write_xlsx(ald_result, path = "ald.xlsx")
```

```
# Filter for significant taxa using we.eBH as the adjusted p-value column

significant_ald_result2 <- ald_result %>%

  filter(wi.eBH < 0.05)

# Reorder the Taxon factor based on the effect size

library(forcats)#no ha fet falta

significant_ald_result2$Taxon <- fct_reorder(significant_ald_result2$Taxon,
significant_ald_result2$effect)


#export o excel

write_xlsx(significant_ald_result2, path = "ald_significant2_family.xlsx")


#bar plot

ggplot(significant_ald_result2, aes(x = Taxon, y = effect, fill = Taxon)) +

  geom_bar(stat = "identity", width = 0.7) + # Adjusted width

  labs(x = "Family", y = "Effect Size") +

  theme(

    axis.text.x = element_text(angle = 90, vjust = 0.5, hjust = 1, size = 6.5), # Adjusted x-axis text
size

    axis.text.y = element_text(size = 6), # Adjusted y-axis text size

    plot.margin = margin(t = 20, r = 20, b = 20, l = 20), # Adjusted margins

    legend.position = "none" # Remove the legend

  )


#Volcano Plot

# Assuming ald_result includes columns like 'effect' and 'we.eBH'

ggplot(significant_ald_result2, aes(x = effect, y = -log10(wi.eBH), color = Taxon)) +

  geom_point(size = 2) +

  labs(x = "Effect Size", y = "-log10(Adjusted p-value)", color = "Taxon") +

  geom_hline(yintercept = -log10(0.05), linetype = "dashed", color = "red") +  # Add significance
threshold

  geom_vline(xintercept = 0, linetype = "dashed", color = "black") +  # Add effect size threshold

  theme_bw() +
```

```
 theme(
  legend.text = element_text(size = 5), # Further reduce legend text size
  legend.key.size = unit(0.4, "lines"), # Further reduce legend key size
  legend.title = element_text(size = 4), # Adjust legend title size
  legend.position = "bottom", # Move legend to the bottom
  legend.box.spacing = unit(0.2, "lines"), # Adjust the spacing around the legend box
  legend.spacing.x = unit(0.2, "lines") # Adjust the spacing between legend items
 )

###PHYLUM LEVEL

# Assuming pseq_family_filt is your phyloseq object and site is your grouping variable
sample_data1 <- data.frame(sample_data(pseq_family_filt))
count_data1 <- as.matrix(otu_table(pseq_family_filt))
taxonomy_data <- as.data.frame(tax_table(pseq_family_filt))

# Ensure that Phylum is a character column
taxonomy_data$Phylum <- as.character(taxonomy_data$Phylum)

# Convert count_data to a data frame and merge with taxonomy_data
count_data_df <- as.data.frame(count_data1) %>%
 rownames_to_column("OTU")

# Merge count data with taxonomy data
merged_data <- taxonomy_data %>%
 rownames_to_column("OTU") %>%
 left_join(count_data_df, by = "OTU")

# Aggregate count data to the phylum level
phylum_data <- merged_data %>%
 group_by(Phylum) %>%
```

```
  summarise(across(where(is.numeric), sum, na.rm = TRUE))

# Convert phylum_data to matrix form for ALDEx2
phylum_counts <- as.matrix(phylum_data[,-1])
rownames(phylum_counts) <- phylum_data$Phylum

# Subset data for specific pairwise comparison
site_subset1 <- subset(sample_data1, site %in% c("wild", "captive"))
count_data_subset1 <- phylum_counts[, colnames(phylum_counts) %in%
rownames(site_subset1)]

# Perform ALDEx2 analysis on subset
ald_result1 <- aldex(count_data_subset1, site_subset1$site, denom = "all", mc.samples = 295)

# Add Phylum names to ald_result
ald_result1$Taxon <- rownames(ald_result1)

# Filter for significant taxa using we.eBH as the adjusted p-value column
significant_ald_result3 <- ald_result1 %>%
  filter(wi.eBH < 0.05)

# Reorder the Taxon factor based on the effect size
significant_ald_result3$Taxon <- fct_reorder(significant_ald_result3$Taxon,
significant_ald_result3$effect)

# Bar plot
ggplot(significant_ald_result3, aes(x = Taxon, y = effect, fill = Taxon)) +
  geom_bar(stat = "identity", width = 0.7) + # Adjusted width
  labs(x = "Phylum", y = "Effect Size") +
  theme(
    axis.text.x = element_text(angle = 90, vjust = 0.5, hjust = 1, size = 12), # Adjusted x-axis text
size
```

```
  axis.text.y = element_text(size = 6), # Adjusted y-axis text size
  plot.margin = margin(t = 20, r = 20, b = 20, l = 20), # Adjusted margins
  legend.position = "none" # Remove the legend
 )

# Export results to Excel
write_xlsx(significant_ald_result3, path = "significant_ald_phylum3.xlsx")

#CORE MICROBIOME

#See also related functions for the analysis of rare and variable taxa (rare_members; rare_abundance; rare_members; rare_abundance; low_abundance).
library("devtools")
#Prevalence of taxonomic groups
#Relative population frequencies; at 1% compositional abundance threshold:
head(prevalence(gen_rel_abund, detection = 1/100, sort = TRUE))
#Absolute population frequencies (sample count):
head(prevalence(gen_rel_abund, detection = 1/100, sort = TRUE, count = TRUE))
#Core microbiota analysis
#If you only need the names of the core taxa, do as follows. This returns the taxa that exceed the given prevalence and detection thresholds.
core.taxa.standard <- core_members(gen_rel_abund, detection = 0, prevalence = 50/100)
write.csv(core.taxa.standard, file = "gen_core_taxa.csv", row.names = FALSE)
#A full phyloseq object of the core microbiota is obtained as follows:
pseq.core <- core(gen_rel_abund, detection = 0, prevalence = .5)
#We can also collapse the rare taxa into an "Other" category
pseq.core2 <- aggregate_rare(gen_rel_abund, "Genus", detection = 0, prevalence = .5)
#Retrieving the core taxa names from the phyloseq object:
core.taxa <- taxa(pseq.core)
write.csv(core.taxa, file = "gen_core_taxanames.csv", row.names = FALSE)
#Core abundance and diversity
#Total core abundance in each sample (sum of abundances of the core members):
```

```
core.abundance <- sample_sums(core(gen_rel_abund, detection = .01, prevalence = .95))


#Core visualization

#Core line plots

#Determine core microbiota across various abundance/prevalence thresholds with the blanket analysis (Salonen et al. CMI, 2012) based on various signal and prevalences.

# With compositional (relative) abundances

det <- c(0, 0.1, 0.5, 2, 5, 20)/100

prevalences <- seq(.05, 1, .05)

#ggplot(d) + geom_point(aes(x, y)) + scale_x_continuous(trans="log10", limits=c(NA,1))

plot_core(gen_rel_abund,

        prevalences = prevalences,

        detections = det,

        plot.type = "lineplot") +

 xlab("Relative Abundance (%)")


#Core heatmaps

#This visualization method has been used for instance in Intestinal microbiome landscaping: Insight in community assemblage and implications for microbial modulation strategies. Shetty et al. FEMS Microbiology Reviews fuw045, 2017.

#Note that you can order the taxa on the heatmap with the taxa.order argument.

# Core with compositionals:

library(RColorBrewer)

library(reshape)


prevalences <- seq(.05, 1, .05)


detections <- round(10^seq(log10(0.01), log10(.2), length = 9), 3)


# Also define gray color palette

gray <- gray(seq(0,1,length=5))
```

```
#Added pseq.rel, I thin... must be checked if it was in the the rednred version,; where it is initialized

#pseq.rel<- microbiome::transform(pseq, 'compositional')

#min-prevalence gets the 100th highest prevalence

p <- plot_core(gen_rel_abund,

        plot.type = "heatmap",

        colours = NULL,

        prevalences = prevalences,

        detections = detections,

        min.prevalence = prevalence(gen_rel_abund, sort = TRUE)[100]) +

 labs(x = "Detection Threshold\n(Relative Abundance (%))") +


 #Adjusts axis text size and legend bar height

 theme(axis.text.y= element_text(size=8, face="italic"),

    axis.text.x.bottom=element_text(size=8),

    axis.title = element_text(size=10),

    legend.text = element_text(size=8),

    legend.title = element_text(size=10))


print(p)


# Core with absolute counts and horizontal view:

# and minimum population prevalence (given as percentage)

detections <- seq(from = 10, to = round(max(abundances(gen_rel_abund))/10, -1), by = 100)

p <- plot_core(fam_rel_abund, plot.type = "heatmap",

        prevalences = prevalences,

        detections = detections,

        colours = rev(brewer.pal(5, "Spectral")),

        min.prevalence = .1, horizontal = TRUE) +

 theme(axis.text.x= element_text(size=8, face="italic", hjust=1),

    axis.text.y= element_text(size=8),
```

axis.title = element_text(size=10),

legend.text = element_text(size=8),

legend.title = element_text(size=10))

print(p)

#Shared core with venn diagram

#In may instances one would wish to compare core taxa that are shared between multiple groups. Here, we demonstrate how this can be achieved by microbiome and eulerr. The test data is stored in the microbiomeutilities R package and the original source of data is Zackular et al., 2014: The Gut Microbiome Modulates Colon Tumorigenesis.

#Load packages.]

#install.packages("eulerr") # If not installed

library(eulerr)

library(microbiome)

devtools::install_github('microsud/microbiomeutilities')

library(microbiomeutilities)

#We will aim to identify shared core taxa between group variables.

simple way to count number of samples in each group

table(meta(fam_rel_abund)$species.name, useNA = "always")

table(meta(fam_rel_abund)$site, useNA = "always")

table(meta(fam_rel_abund)$study.name, useNA = "always")

#Make a list of species.

species <- unique(as.character(meta(fam_rel_abund)$species.name))

print(species)

#Write a for loop to go through each of the disease_states one by one and combine identified core taxa into a list.

list_core <- c() # an empty object to store information

for (n in species){ # for each variable n in species

 #print(paste0("Identifying Core Taxa for ", n))

```
  ps.sub <- subset_samples(fam_rel_abund, species.name == n) # Choose sample from DiseaseState by n


  core_m <- core_members(ps.sub, # ps.sub is phyloseq selected with only samples from g

                  detection = 0.001, # 0.001 in atleast 90% samples

                  prevalence = 0.75)

  print(paste0("No. of core taxa in ", n, " : ", length(core_m))) # print core taxa identified in each DiseaseState.

  list_core[[n]] <- core_m # add to a list core taxa for each group.

  #print(list_core)

}


print(list_core)


# Specify colors and plot venn

# supplying colors in the order they appear in list_core

mycols <- c(francois_langur="#d6e2e9", udzunga_red_colobus_monkey="#cbf3f0", red_shanked_douc="#fcf5c7",  eastern_black_and_white_colobus="dodgerblue2", silvered-leaf_langur="maroon", tufted_gray_langur="green4", purple_faced_langur="#6A3D9A", golden_snub-nosed_monkey="#FF7F00",  ursine_colobus="gold1", king_colobus="#FB9A99", olive_colobus="darkturquoise", upper_guinea_red_colobus="#CAB2D6")

plot(venn(list_core),

    fills = mycols)

c16 <- c("dodgerblue2", "#E31A1C", "green4", "#6A3D9A",

       "#FF7F00",  "gold1", "#FB9A99", "darkturquoise",

       "#CAB2D6", "#FDBF6F","gray70", "khaki2",

       "maroon", "deeppink1", "blue1", "steelblue4")


#Venn for more than 5 sets example

library(venneuler)
```

H=list('francois langur'=c("Eggerthellaceae", "Muribaculaceae", "Prevotellaceae", "Rikenellaceae","Bacteria_Cyanobacteria_Oxyphotobacteria_Chloroplast_", "Christensenellaceae", "Clostridiales vadinBB60 group", "Eubacteriaceae", "Family XIII", "Lachnospiraceae", "Peptococcaceae", "Peptostreptococcaceae", "Ruminococcaceae", "Spirochaetaceae", "Bacteria_Tenericutes_Mollicutes_Mollicutes RF39_","Bacteria_Tenericutes_Mollicutes_Mollicutes RF39_uncultured bacterium", "Akkermansiaceae"),

'udzungwa red colobus monkey'=c("Bacteroidaceae", "Marinifilaceae", "Muribaculaceae", "p-251-o5", "Prevotellaceae", "Rikenellaceae", "Bacteria_Cyanobacteria_Melainabacteria_Gastranaerophilales_uncultured bacterium", "Bacteria_Firmicutes_Clostridia_Clostridiales_", "Christensenellaceae", "Clostridiales vadinBB60 group", "Family XIII", "Lachnospiraceae", "Peptococcaceae", "Ruminococcaceae", "Erysipelotrichaceae", "Spirochaetaceae", "Bacteria_Tenericutes_Mollicutes_EMP-G18_uncultured bacterium", "Bacteria_Tenericutes_Mollicutes_Izimaplasmatales_uncultured bacterium", "Bacteria_Tenericutes_Mollicutes_Mollicutes RF39_", "Bacteria_Tenericutes_Mollicutes_Mollicutes RF39_uncultured bacterium", "Puniceicoccaceae"),

'red shanked douc'=c("Christensenellaceae", "Lachnospiraceae", "Ruminococcaceae", "Bacteria_Tenericutes_Mollicutes_Mollicutes RF39_", "Akkermansiaceae"),

'tufted gray langur'=c("Methanomethylophilaceae", "Prevotellaceae", "Rikenellaceae", "Christensenellaceae", "Clostridiales vadinBB60 group", "Lachnospiraceae", "Peptococcaceae", "Ruminococcaceae", "Spirochaetaceae", "Bacteria_Tenericutes_Mollicutes_Mollicutes RF39_", "Bacteria_Tenericutes_Mollicutes_Mollicutes RF39_uncultured bacterium"),

'purple faced langur'=c("Clostridiales vadinBB60 group", "Lachnospiraceae", "Ruminococcaceae", "Bacteria_Tenericutes_Mollicutes_Izimaplasmatales_uncultured bacterium", "Bacteria_Tenericutes_Mollicutes_Mollicutes RF39_","Bacteria_Tenericutes_Mollicutes_Mollicutes RF39_uncultured bacterium"),

'golden snub-nosed monkey'=c("Bacteria_Bacteroidetes_Bacteroidia__", "Bacteria_Bacteroidetes_Bacteroidia_Bacteroidales_", "Muribaculaceae", "Flavobacteriaceae","Bacteria_Firmicutes___", "Bacteria_Firmicutes_Clostridia_Clostridiales_", "Lachnospiraceae", "Peptococcaceae", "Ruminococcaceae", "Pseudomonadaceae", "Spirochaetaceae", "Synergistaceae"),

'ursine colobus'=c("Methanomethylophilaceae", "Eggerthellaceae", "Bacteroidaceae", "Marinifilaceae", "Muribaculaceae", "Bacteria_Cyanobacteria_Melainabacteria_Gastranaerophilales_", "Bacteria_Cyanobacteria_Melainabacteria_Gastranaerophilales_uncultured bacterium", "Bacteria_Cyanobacteria_Melainabacteria_Gastranaerophilales_uncultured rumen bacterium", "Bacteria_Cyanobacteria_Oxyphotobacteria_Chloroplast_", "Bacteria_Firmicutes_Clostridia_Clostridiales_", "Christensenellaceae", "Clostridiales vadinBB60 group", "Family XIII", "Lachnospiraceae", "Peptococcaceae", "Ruminococcaceae", "Veillonellaceae", "Pirellulaceae", "Spirochaetaceae", "Bacteria_Tenericutes_Mollicutes_Izimaplasmatales_uncultured bacterium", "Bacteria_Tenericutes_Mollicutes_Mollicutes RF39_"),

```
'king colobus'=c("Methanobacteriaceae", "Bacteroidaceae", "Marinifilaceae",
"Muribaculaceae", "p-2534-18B5 gut group", "Prevotellaceae", "Rikenellaceae",
"Bacteria_Cyanobacteria_Melainabacteria_Gastranaerophilales_",
"Bacteria_Cyanobacteria_Melainabacteria_Gastranaerophilales_uncultured bacterium",
"Bacteria_Cyanobacteria_Melainabacteria_Gastranaerophilales_uncultured rumen bacterium",
"Bacteria_Firmicutes_Clostridia_Clostridiales_", "Christensenellaceae", "Clostridiaceae 1",
"Clostridiales vadinBB60 group", "Family XIII", "Lachnospiraceae", "Peptococcaceae",
"Ruminococcaceae", "Pirellulaceae", "Burkholderiaceae", "Spirochaetaceae",
"Bacteria_Tenericutes_Mollicutes_Izimaplasmatales_uncultured bacterium",
"Bacteria_Tenericutes_Mollicutes_Mollicutes RF39_",
"Bacteria_Tenericutes_Mollicutes_Mollicutes RF39_uncultured bacterium",
"Bacteria_Tenericutes_Mollicutes_Mollicutes RF39_uncultured prokaryote" ),

'olive colobus'=c("Methanomethylophilaceae",
"Bacteria_Bacteroidetes_Bacteroidia_Bacteroidales_", "Bacteroidaceae", "F082",
"Marinifilaceae", "Muribaculaceae", "p-251-o5", "Prevotellaceae", "Rikenellaceae",
"Bacteria_Bacteroidetes_Bacteroidia_Bacteroidales_uncultured",
"Bacteria_Cyanobacteria_Melainabacteria_Gastranaerophilales_",
"Bacteria_Cyanobacteria_Melainabacteria_Gastranaerophilales_uncultured bacterium",
"Bacteria_Cyanobacteria_Melainabacteria_Gastranaerophilales_uncultured rumen bacterium",
"Campylobacteraceae", "Bacteria_Firmicutes_Clostridia_Clostridiales_",
"Christensenellaceae", "Clostridiales vadinBB60 group", "Family XIII", "Lachnospiraceae",
"Peptococcaceae", "Ruminococcaceae",
"Bacteria_Proteobacteria_Alphaproteobacteria_Rhodospirillales_uncultured",
"Bacteria_Proteobacteria_Alphaproteobacteria_Rickettsiales_uncultured", "Spirochaetaceae",
"Bacteria_Tenericutes_Mollicutes_Izimaplasmatales_uncultured bacterium",
"Bacteria_Tenericutes_Mollicutes_Mollicutes RF39_",
"Bacteria_Tenericutes_Mollicutes_Mollicutes RF39_uncultured rumen bacterium",
"Puniceicoccaceae"),

'upper guinea red colobus'=c("Bacteria_Bacteroidetes_Bacteroidia__", "Bacteroidaceae", "p-
251-o5", "Prevotellaceae", "Rikenellaceae",
"Bacteria_Bacteroidetes_Bacteroidia_Bacteroidales_uncultured",
"Bacteria_Cyanobacteria_Melainabacteria_Gastranaerophilales_uncultured bacterium",
"Campylobacteraceae", "Clostridiales vadinBB60 group", "Family XIII", "Lachnospiraceae",
"Peptococcaceae", "Ruminococcaceae", "Erysipelotrichaceae",
"Bacteria_Kiritimatiellaeota_Kiritimatiellae_WCHB1-41_uncultured bacterium",
"Bacteria_Proteobacteria_Alphaproteobacteria_Rhodospirillales_uncultured",
"Enterobacteriaceae", "Spirochaetaceae",
"Bacteria_Tenericutes_Mollicutes_Izimaplasmatales_uncultured bacterium",
"Bacteria_Tenericutes_Mollicutes_Mollicutes RF39_",
"Bacteria_Tenericutes_Mollicutes_Mollicutes RF39_uncultured bacterium",
"Bacteria_Tenericutes_Mollicutes_Mollicutes RF39_uncultured rumen bacterium",
"Akkermansiaceae")

)
```

```r
# Compute Euler data
library(eulerr)
venn_data <- euler(H)

# Install RColorBrewer if not already installed
if (!requireNamespace("RColorBrewer", quietly = TRUE)) install.packages("RColorBrewer")
library(RColorBrewer)

# Generate a color palette
num_species <- length(H)
species_colors <- brewer.pal(min(num_species, 8), "Set3")  # Use Set3 palette (max 8 colors)
# If more than 8 species, repeat or generate additional colors
if (num_species > 8) {
  species_colors <- colorRampPalette(brewer.pal(8, "Set3"))(num_species)
}

# Preview the colors
print(species_colors)

#Match the colors with the species names.
names(species_colors) <- names(H)
print(species_colors)

#plot
plot(venn_data,
     fills = list(fill = species_colors, alpha = 0.5),
     edges = list(col = "black", lwd = 2),
     labels = list(fontsize = 8))

# Add legend
legend("topright",
```

```
      legend = names(H),
      fill = species_colors,
      title = "Species",
      cex = 0.8)

# Find intersection of all species
common_microbiome <- Reduce(intersect, H)

# Print common core microbiome
print(common_microbiome)
#[1] "Lachnospiraceae" "Ruminococcaceae"

#
library(grid)

# Plot the Venn diagram
plot(venn_data,
     fills = list(fill = species_colors, alpha = 0.5),
     edges = list(col = "black", lwd = 2),
     labels = list(fontsize = 8))

# Add legend
legend("topright",
      legend = names(H),
      fill = species_colors,
      title = "Species",
      cex = 0.8)

# Add common microbiome at the center
grid.text(paste(common_microbiome, collapse = "\n"), x = 0.5, y = 0.5, gp = gpar(fontsize = 10,
col = "darkgreen", fontface = "bold"))
```
