# Supplementary material for: Colobine gut microbiome vulnerability to captivity: a meta-analysis of herbivorous primates
Source: Anim Microbiome. 2026 Jul 29;8:110. doi: 10.1186/s42523-026-00600-6 (PMC13715106; doi:10.1186/s42523-026-00600-6)
Supplement: Supplementary file 2 [file 42523_2026_600_MOESM5_ESM.pdf]

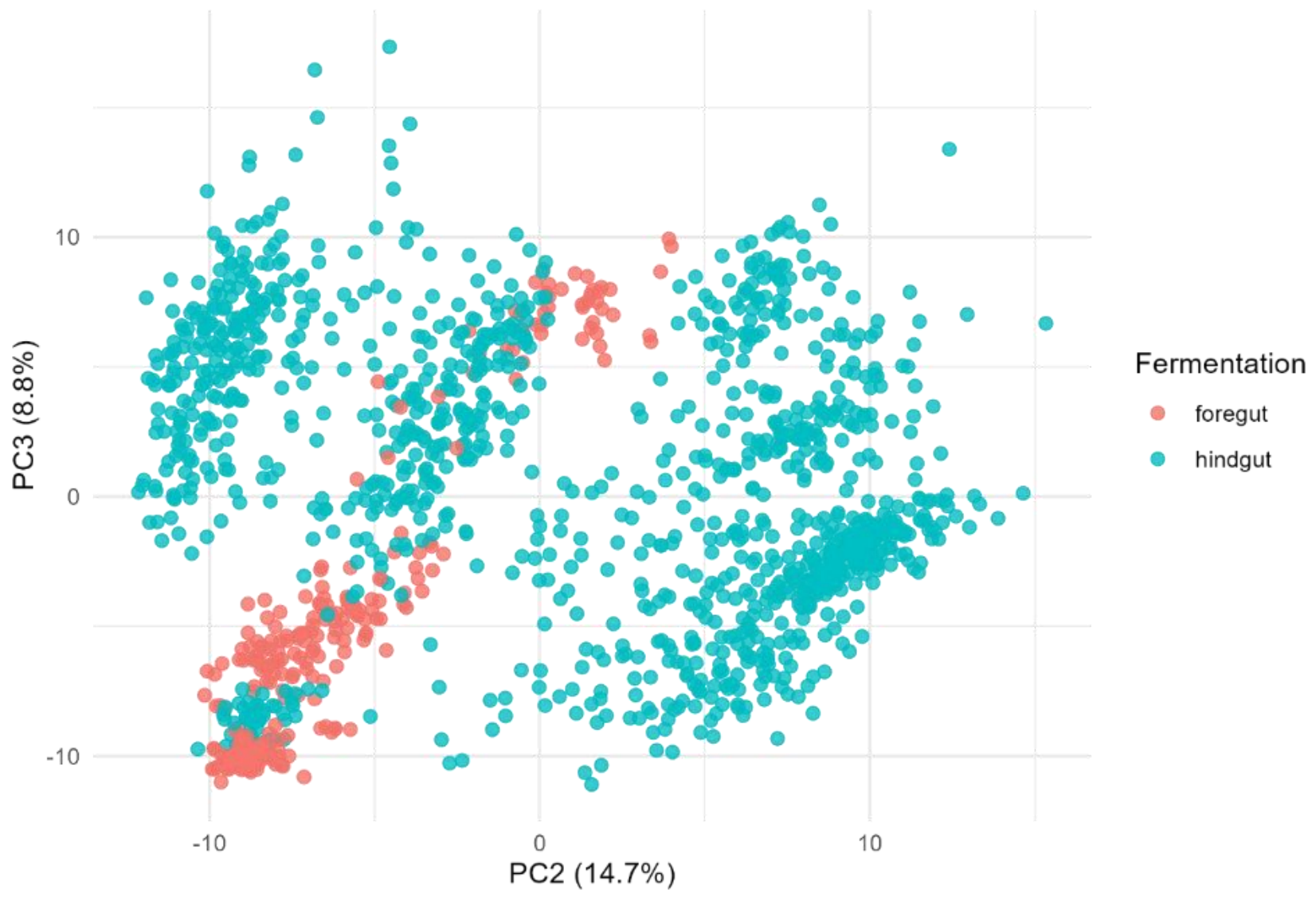

Figure S1. Principal Component Analysis (PCA) of Aitchison distances calculated from CLR-transformed family-level microbiome data for wild foregut- and hindgut-fermenting primates. Samples are coloured by fermentation strategy. This plot shows the PC2–PC3 ordination corresponding to Fig. 2A.

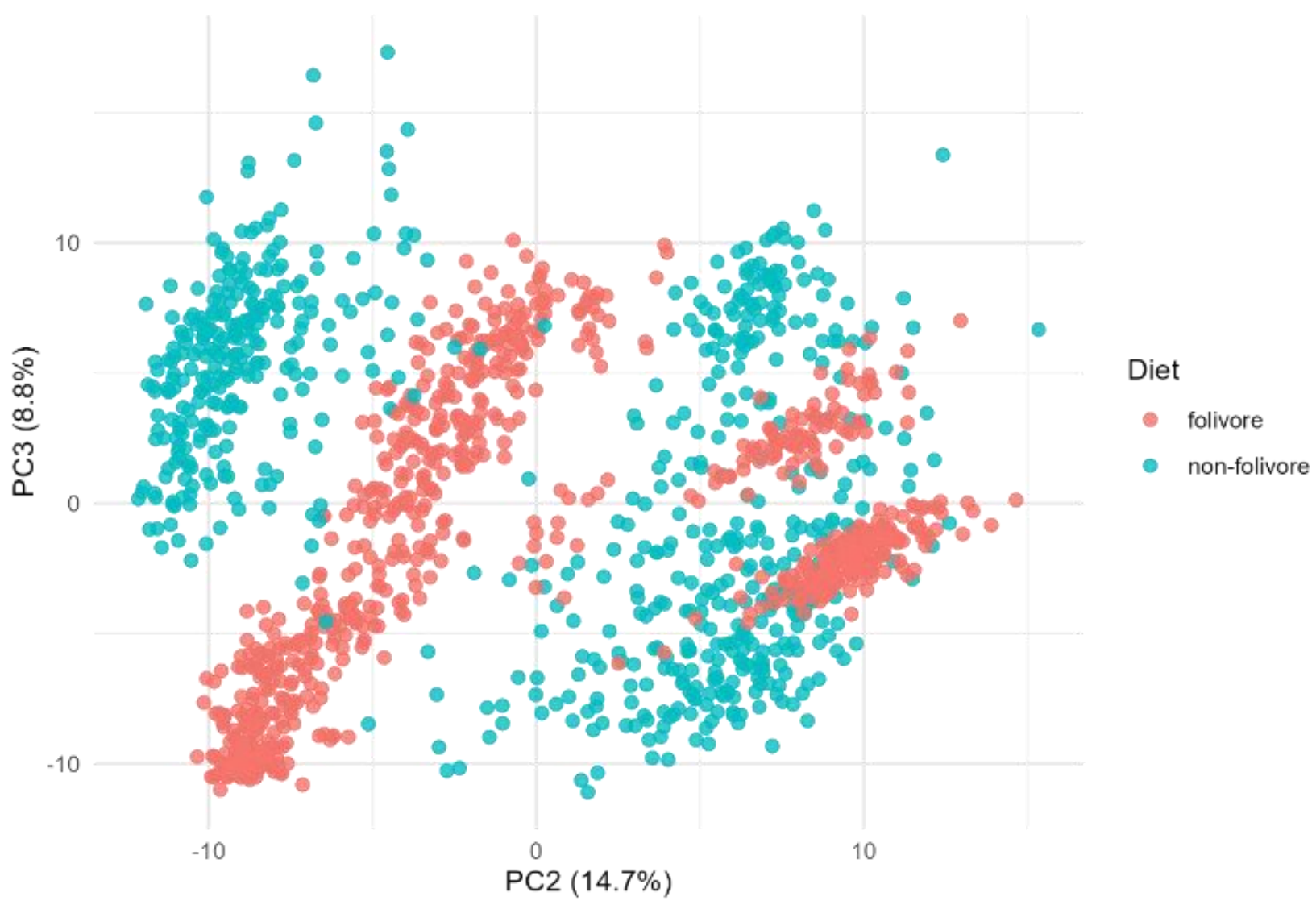

Figure S2. Principal Component Analysis (PCA) of Aitchison distances calculated from CLR-transformed family-level microbiome data for wild folivorous and non-folivorous

primates. Samples are coloured by dietary category. This plot shows the PC2–PC3 ordination corresponding to Fig. 3A.

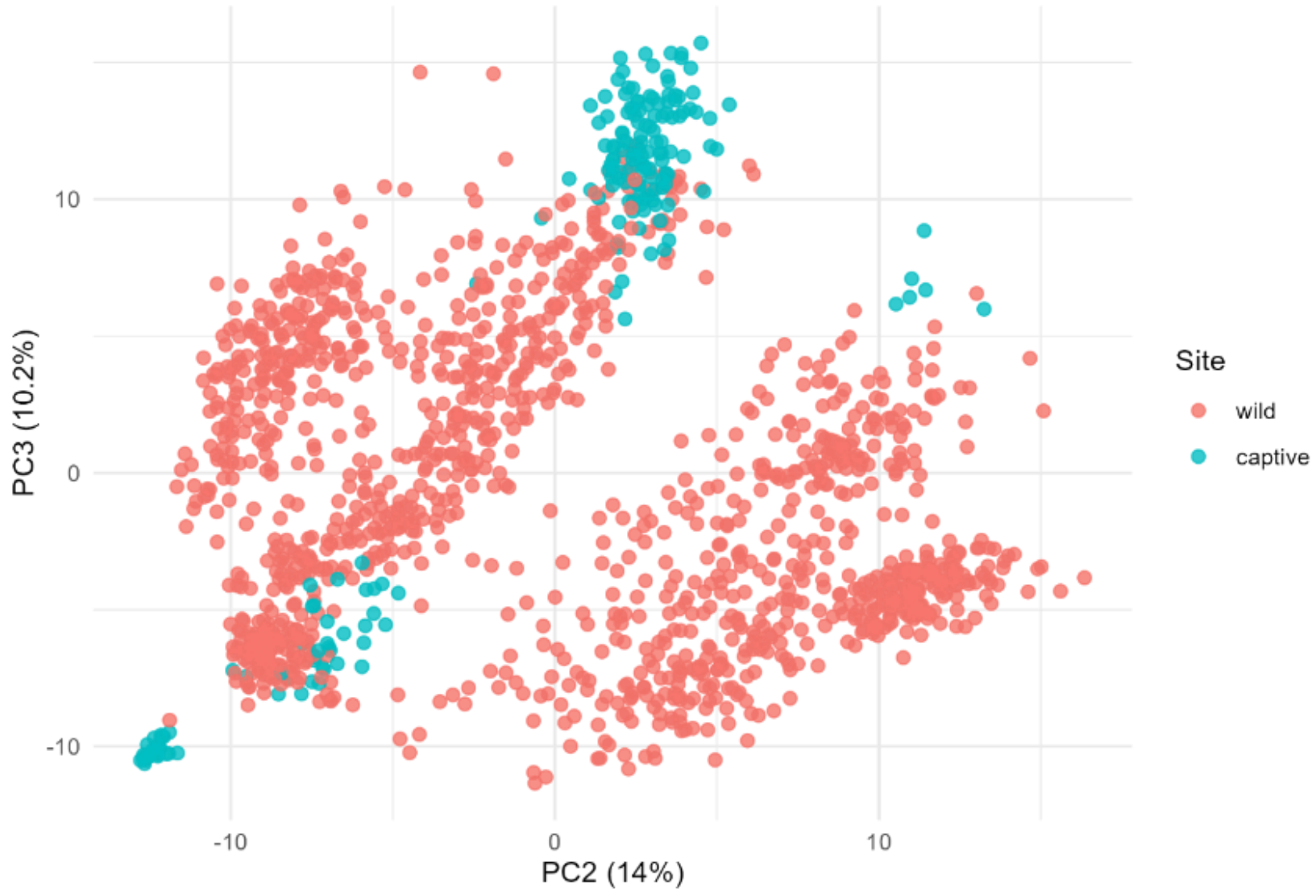

Figure S3. Principal Component Analysis (PCA) of Aitchison distances calculated from CLR-transformed family-level microbiome data for wild folivorous and non-folivorous primates. Samples are coloured by dietary category. This plot shows the PC2–PC3 ordination corresponding to Fig. 3A.

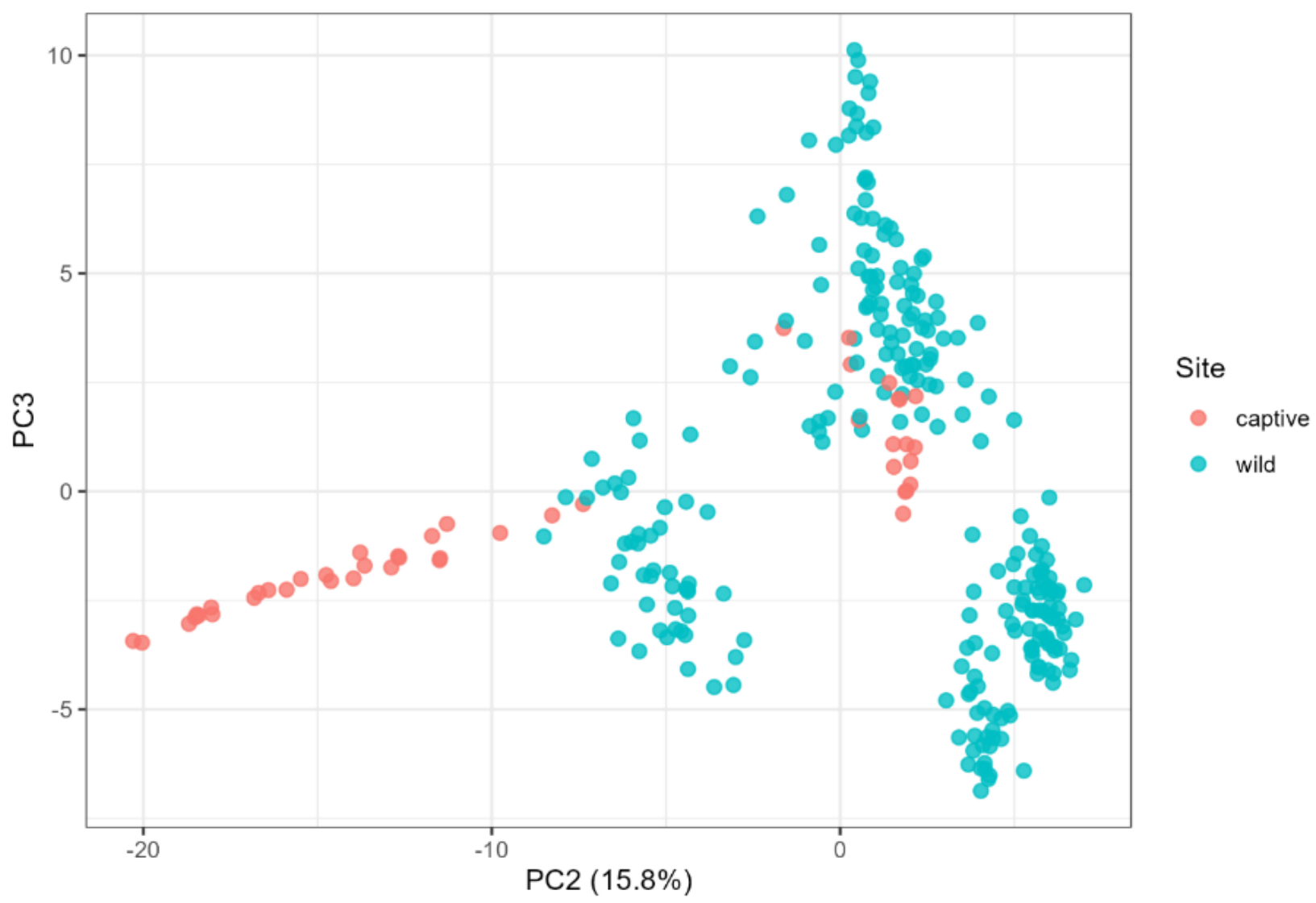

Figure S4. Principal Component Analysis (PCA) of Aitchison distances calculated from CLR-transformed family-level microbiome data for wild and captive colobines. Samples are coloured by site (wild or captive). This plot shows the PC2–PC3 ordination corresponding to Fig. 7A.

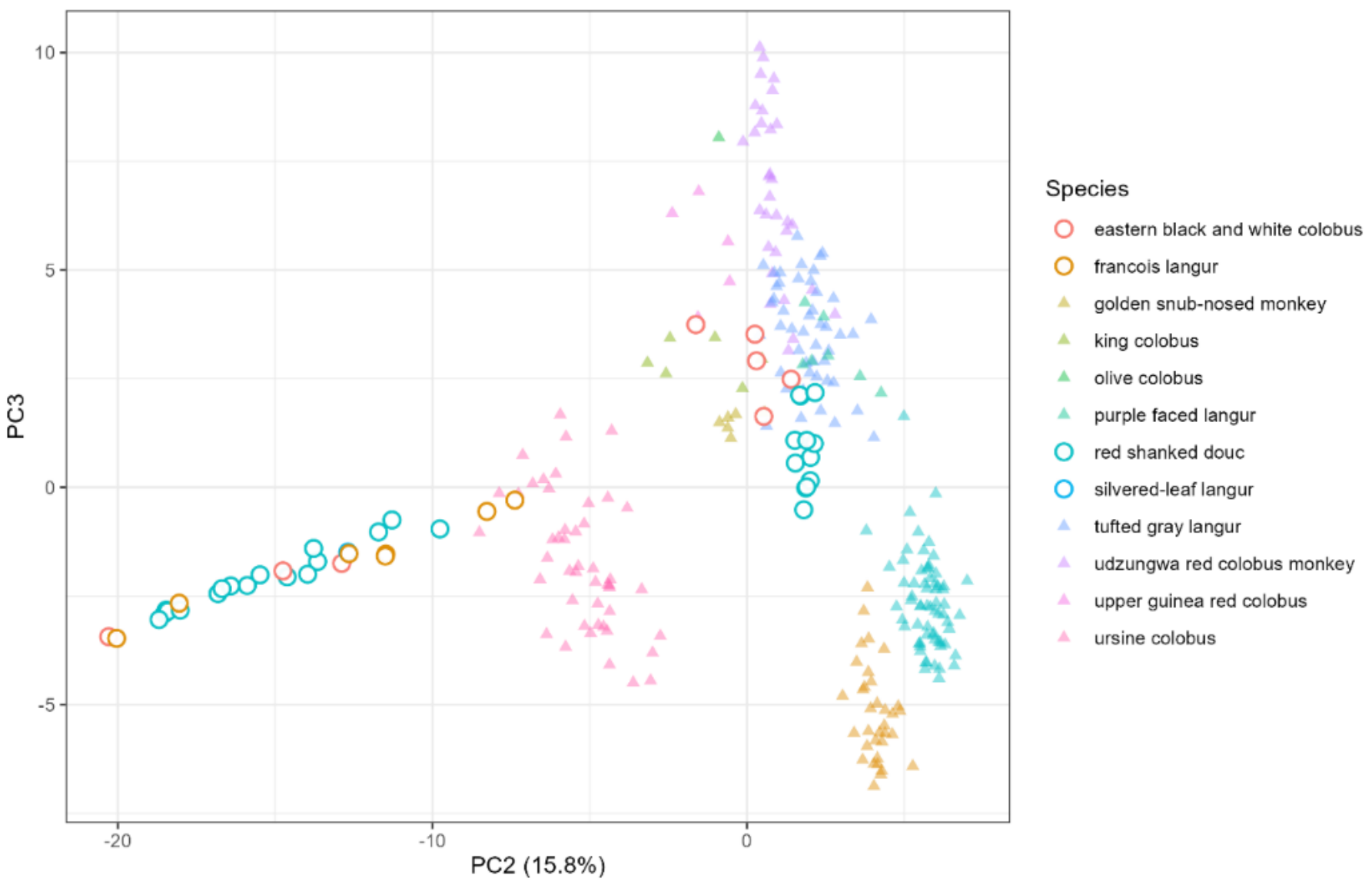

Figure S5. Principal Component Analysis (PCA) of Aitchison distances calculated from CLR-transformed family-level microbiome data for colobines. Samples are coloured by species. This plot shows the PC2–PC3 ordination corresponding to Fig. 7B and illustrates clustering by species independent of captivity status.
